# Autophagy regulates Notch degradation and modulates stem cell development and neurogenesis

**DOI:** 10.1038/ncomms10533

**Published:** 2016-02-03

**Authors:** Xiaoting Wu, Angeleen Fleming, Thomas Ricketts, Mariana Pavel, Herbert Virgin, Fiona M. Menzies, David C. Rubinsztein

**Affiliations:** 1 Department of Medical Genetics, Cambridge Institute for Medical Research (CIMR), University of Cambridge, Cambridge CB2 0XY, UK; 2 Department of Physiology, Development and Neuroscience, University of Cambridge, Downing Street, Cambridge CB2 3EG, UK; 3 Department of Pathology and Immunology, Washington University School of Medicine, St Louis, Missouri 63110, USA

## Abstract

Autophagy is a conserved, intracellular, lysosomal degradation pathway. While mechanistic aspects of this pathway are increasingly well defined, it remains unclear how autophagy modulation impacts normal physiology. It is, however, becoming clear that autophagy may play a key role in regulating developmental pathways. Here we describe for the first time how autophagy impacts stem cell differentiation by degrading Notch1. We define a novel route whereby this plasma membrane-resident receptor is degraded by autophagy, via uptake into ATG16L1-positive autophagosome-precursor vesicles. We extend our findings using a physiologically relevant mouse model with a hypomorphic mutation in *Atg16L1*, a crucial autophagy gene, which shows developmental retention of early-stage cells in various tissues where the differentiation of stem cells is retarded and thus reveal how modest changes in autophagy can impact stem cell fate. This may have relevance for diverse disease conditions, like Alzheimer's Disease or Crohn's Disease, associated with altered autophagy.

## Abstract (web summary)

The molecular mechanisms behind how autophagy may impact on developmental pathways and cell fate decisions are unclear. Here Wu *et al.* identify Notch receptors being taken up into ATG16L1-positive autophagosomes and, using a mouse mutant model, show that changes in autophagy can impact on stem cell fate.

## Graphical abstract

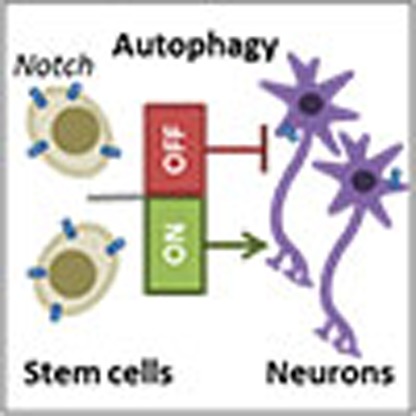

Autophagy is an evolutionarily conserved pathway that delivers substrates to lysosomes for degradation. Autophagosomes are derived from cup-shaped intracytoplasmic structures, called phagophores, which engulf cytoplasmic contents when their edges extend and seal to form double-membraned autophagosomes. Ultimately, autophagosomes fuse with lysosomes, where the autophagic substrates are degraded^1^. Phagophore formation requires PI(3)P, which is produced by VPS34 in a complex with other proteins including Beclin-1. The biogenesis of mammalian autophagosomes also involves two ubiquitin-like molecules, ATG12 and LC3/ATG8 (ref. 2). In the first of these reactions, the C-terminal glycine of ATG12 is conjugated to ATG5. This is a ubiquitin-like conjugation involving ATG7 as the E1-like enzyme and ATG10 as the E2-like enzyme. The ATG12–ATG5 conjugate then forms an 800 kDa complex with ATG16L1 (ref. 3). ATG16L1 localizes to the phagophores, and dissociates from fully formed (mature) autophagosomes. In the second ubiquitin-like reaction, LC3 is conjugated to phosphatidylethanolamine to form lipidated LC3-II by ATG7 and ATG3, as the relevant E1- and E2-like enzymes, respectively. LC3-II is specifically targeted to elongating pre-autophagosomal structures and then remains on mature autophagosomes until after fusion with lysosomes. Thus, the LC3-II level corresponds to the number of autophagosomes and autolysosomes^4^.

Autophagy has key roles in normal physiology and disease, including neurodegenerative conditions, metabolic diseases and cancer^5^. There has been a growing interest in the role of autophagy in embryogenesis and development, as deletion of various *ATG* genes causes overt phenotypes or lethality in mice^6^,^7^,^8^,^9^. Furthermore, autophagy interacts with crucial developmental pathways like Wnt, Sonic hedgehog, transforming growth factor-β and fibroblast growth factor^10^,^11^,^12^,^13^. This suggests that autophagy may regulate cell fate decisions, like differentiation and proliferation, but the mechanisms by which autophagy exerts directional and specific control of development have remained elusive.

Here we tested whether autophagy regulates the Notch pathway, which has important roles in both disease and development. Notch signalling is crucial from embryogenesis to adulthood^14^,^15^. It is considered to be a master regulator of neural stem cells and neuronal development, where it is often used to select between pre-existing developmental signals and pre-empt cell fate decisions^16^. Notch receptors and ligands are both transmembrane proteins. Maturation and activation of Notch requires a number of proteolytic cleavage steps (Supplementary Fig. 1a). During maturation, most Notch1 receptors are cleaved by furin-like convertases to generate the extracellular part (Notch extracellular domain, NECD) and the transmembrane—intracellular part (Notch transmembrane domain, NTMD)—which are non-covalently linked. This is referred to as the S1 cleavage, and it enables the receptor to be activated by the ligand. At the plasma membrane, the first cleavage is at the extracellular site (site 2/S2) located 12 amino acids before the transmembrane domain and is mediated by ADAM-family metalloproteases. The membrane-tethered intermediate form created is referred to as Notch extracellular truncation. Notch extracellular truncation is then cleaved by the γ-secretase within the transmembrane domain at sites 3 (inner plasma membrane leaflet) and 4. After the second cleavage, the Notch intracellular domain (NICD) is released into the cytosol and translocates into the nucleus to bind the transcription factor CSL and its coactivator Mastermind (Mam), which promote transcription of Notch target genes, mainly from the Hes family^14^,^16^.

In this study, we examined whether Notch signalling is regulated by autophagy in mammalian cells and how this occurs. We investigated if autophagy-defective mice had Notch-dependent phenotypes. In our model, autophagy regulates Notch degradation, which correlates with the expected consequences of Notch hyperactivity on stem cell development and neurogenesis.

## Results

### Autophagic activity impacts Notch signalling

**Figure 1:**
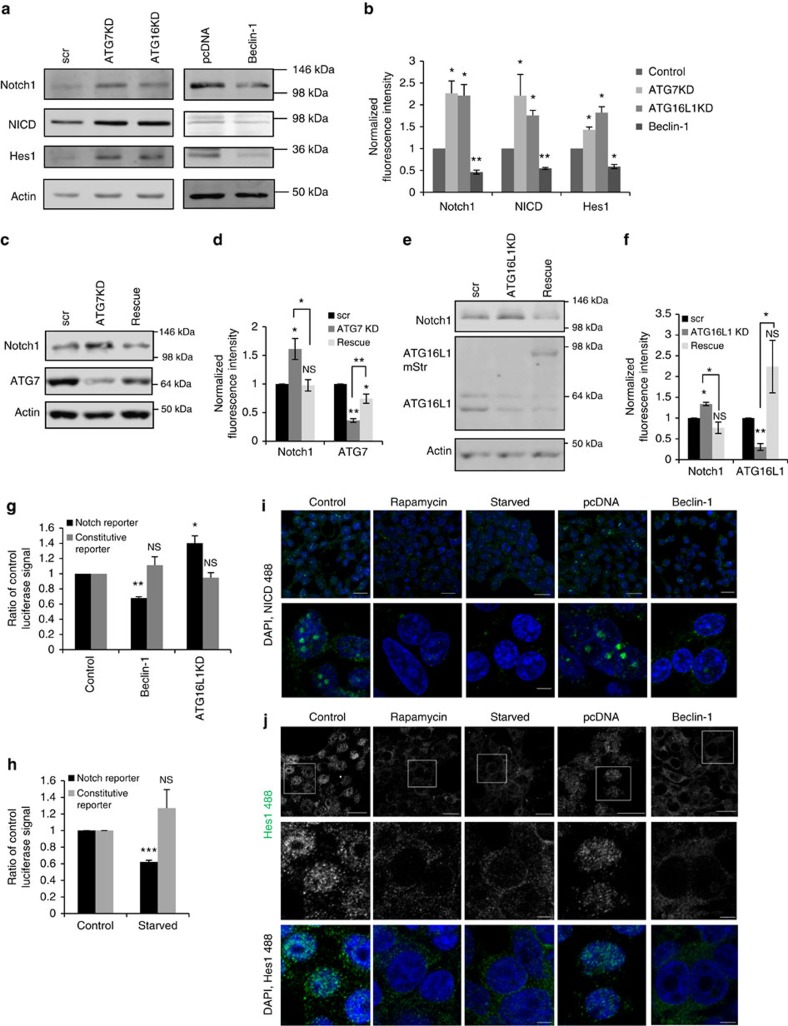
Autophagy modulates Notch signalling pathway. (**a**) Representative western blot showing levels of Notch1 and its downstream effectors. Autophagy was inhibited in HEK cells by Smartpool siRNA (KD) for ATG7 or ATG16L1 or activated by pcDNA/Beclin-Flag transfection. Scr=scrambled siRNA. Actin is loading control. (**b**) Quantification of western blots for Notch1 and effectors, relative to actin, normalized for relevant control. *P<0.05 or **P<0.01 by paired *t*-test. *n*=3 in triplicates. Error bars=s.e.m. (**c**) Representative western blot showing effect of ATG7 Smartpool siRNA knockdown and ATG7-wt cDNA rescue on Notch1 levels. (**d**) Quantification of western blots for Notch1 and ATG7, relative to actin, normalized for relevant control. *P<0.05 or **P<0.01 by paired *t*-test. NS denotes not significant. *n*=4. Error bars=s.e.m. (**e**) Representative western blot showing the effect of ATG16L1 Smartpool siRNA knockdown and ATG16L1 mStr rescue on Notch1 levels. (**f**) Quantification of western blots for Notch1 and ATG16L1/ATG16L1 mStr, relative to actin, normalized for relevant control. *P<0.05 or **P<0.01 by paired *t*-test. *n*=3. Error bars=s.e.m. (**g**,**h**) HEK cells were transfected with control (scrambled) siRNA/ATG16L1 siRNA +Dll1 ligands (ATG16L1KD), or pcDNA/Beclin-Flag. Notch pathway activity was measured by firefly luciferase reporter assay with RBP-Jκ coupled reporter using Renilla luciferase with constitutive promoter as control. Ratio of sample to control is shown. *P<0.05 and **P<0.01. NS denotes not significant by unpaired *t-*test. *n*=3. Error bars=s.e.m. (**i**) Effect of autophagy on the nuclear translocation of NICD. Scale bar, 20 μm for images of the upper panel. Scale bar, 5 μm for all lower panel images. (**j**) HEK cells were treated with rapamycin/starved with HBSS, or transfected with pcDNA/Beclin-1. Magnifications of areas in white boxes are shown in lower images and with DAPI in lower panels. Scale bar, 10 μm for upper panels. Scale bar, 5 μm for bottom two rows.

Depletion of the levels of the core autophagy proteins ATG7 and ATG16L1 by small interfering RNA (siRNA) knockdown using Smartpools as well as individual deconvoluted oligonucleotides, inhibits autophagy, indicated by reduced LC3-II levels^17^, among other readouts (Supplementary Fig. 1b, c). We observed that ATG7 or ATG16L1 knockdown caused elevation of the levels of Notch1, as well as the activated, cleaved form of Notch, NICD and the protein levels of its target gene, Hes1 (Fig. 1a,b). Since the canonical degradation pathway for Notch1 is via endocytosis, it is important to note that ATG16L1 knockdown does not impair endocytosis^18^. The increase in Notch1 level after KD was rescued by overexpression of the relevant target protein (Fig. 1c–f). Conversely, Beclin-1 overexpression, which enhances autophagosome formation (Supplementary Fig. 2a), reduced the levels of Notch1, NICD and Hes1 (Fig. 1a,b). Consistent with the genetic data, Notch1, NICD and Hes1 levels were also reduced by rapamycin or starvation, known autophagy stimuli (Supplementary Fig. 2b,c). While the levels of Notch1 and its downstream effectors responded to changes in autophagy, levels of the Notch ligand, Dll1 (ref. 16), were unaltered by these genetic manipulations (Supplementary Fig. 2d).

These results suggest that autophagy modulation is able to alter Notch signalling. To confirm these functional effects, we used an RBP-Jκ luciferase assay, which responds to a transcription factor downstream of Notch signalling. The pathway was significantly inhibited by Beclin-1 overexpression but activated by ATG16L1 knockdown (Fig. 1g) and the magnitude of these changes was similar to those previously described with other perturbations of the Notch pathway^19^. The pathway was also inhibited under starvation conditions (Fig. 1h). When Notch signalling is active, NICD translocates to the nucleus and promotes the transcription of Hes1. Consistent with the luciferase assay data, the nuclear localizations of NICD and Hes1 were ablated and the overall level of their staining decreased when cells were exposed to autophagy inducers, rapamycin or starvation, or overexpression of Beclin-1 (Fig. 1i,j).

**Figure 2:**
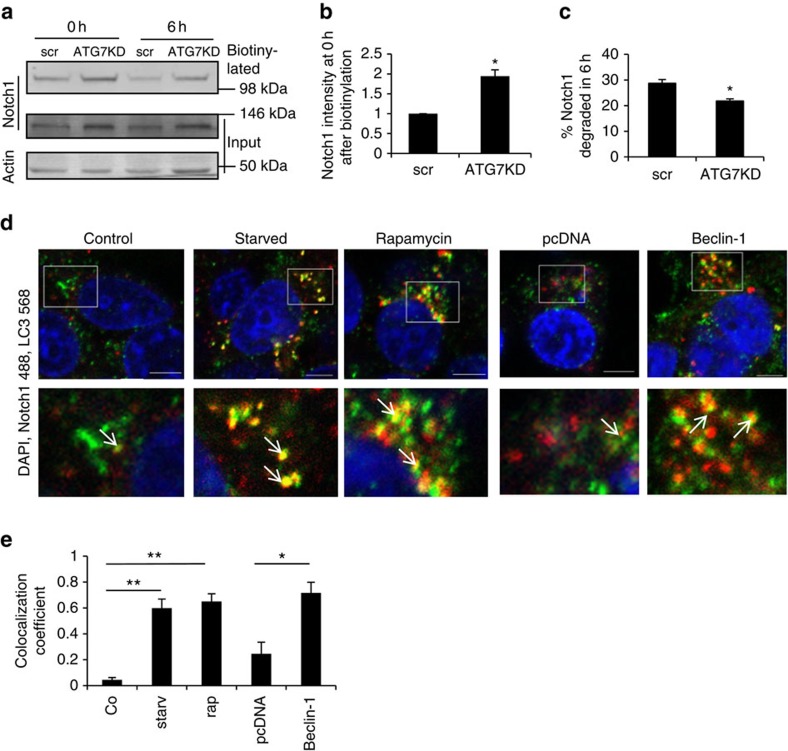
Autophagy regulates Notch1 degradation. (**a**) Effect of autophagy inhibition on degradation of Notch1 was assessed by biotinylation assay in HEK cells treated with ATG7 siRNA (ATG7KD). The cell surface proteins were biotinylated and cells were immediately collected or incubated at 37 °C for 6 h to allow internalization and degradation. After lysis, biotinylated proteins were pulled down with streptavidin beads and the amount of biotinylated Notch1 remaining assessed by western blot (top panel). Western blots for total Notch1 levels and actin levels in the input are shown as controls. (**b**) Quantification of surface Notch1 at time point 0 h after autophagy inhibition. *P<0.05 by paired *t*-test. *n*=3. Error bars=s.e.m. (**c**) Quantification of Notch1 degradation rate. The ratio of 6 h/0 h biotinylated Notch1 level is shown for scrambled siRNA (scr) and ATG7KD samples. *P<0.05 by unpaired *t*-test. *n*=3. Error bars=s.e.m. (**d**) HEK cells were starved with HBSS (2 h) or treated with DMSO (control) /rapamycin or transfected with pcDNA or Beclin-Flag (2 days). Cells were immunostained for Notch1 (green) and LC3 (red) and stained with DAPI (blue). The white box indicates the location of the enlargement. The arrows show LC3 and Notch1 colocalization. Scale bar, 5 μm (**e**) Colocalization of Notch1 with LC3 from 2 days (starv=starved, rap=rapamycin). Mander's colocalization coefficient was measured using Volocity software. Unpaired *t*-test **P<0.01. *P<0.05. *n*=3. Error bars=s.e.m.

To identify the mechanism whereby autophagy modulation is able to impact on Notch levels and signalling, we first considered the role of autophagy in Notch1 degradation. We confirmed that modulations in autophagy are able to alter Notch1 levels on the plasma membrane where it is able to bind its ligand and be activated. We performed biotinylation experiments at 4 °C to label cell surface proteins and observed that ATG7 siRNA knockdown resulted in an increase in the levels of Notch1 on the plasma membrane (Fig. 2a,b). To test if autophagy modulated Notch1 degradation directly we performed pulse and chase experiments by biotin labelling the cell surface proteins then incubating cells for 6 h at 37 °C to allow internalization and degradation of the labelled protein pool. The rate of cell surface Notch1 degradation in ATG7 knockdown cells was 1/3 slower than in the control cells (Fig. 2a,c), suggesting that autophagy is able to directly degrade Notch1.

If Notch1 is degraded by autophagy, then it should be localized to autophagosomes, which can be labelled with LC3. Under basal conditions, Notch1 can clearly be seen to localize to the plasma membrane by immunocytochemistry (Supplementary Fig. 3a). However, Notch1 can also be observed intracellularly, where a small fraction can be seen to colocalize with LC3 (Fig. 2d, Supplementary Fig. 3b). When cells are exposed to autophagy stimuli like starvation, rapamycin and Beclin-1 overexpression, the Notch1-LC3 colocalization is increased compared with control conditions (Fig. 2d,e). In keeping with our observation that Dll1 levels did not change on autophagy induction, it did not colocalize with LC3 even on autophagy stimulation (Supplementary Fig. 3c,d). Likewise, we detected no colocalization of NICD with LC3 under basal or autophagy-enhanced conditions (Supplementary Fig. 3e,f). This indicates that the decreased level of NICD observed on autophagy induction is likely to be a consequence of Notch1 degradation, rather than direct autophagic clearance of NICD.

### Notch1 can enter autophagosomes independent from endocytosis

**Figure 3:**
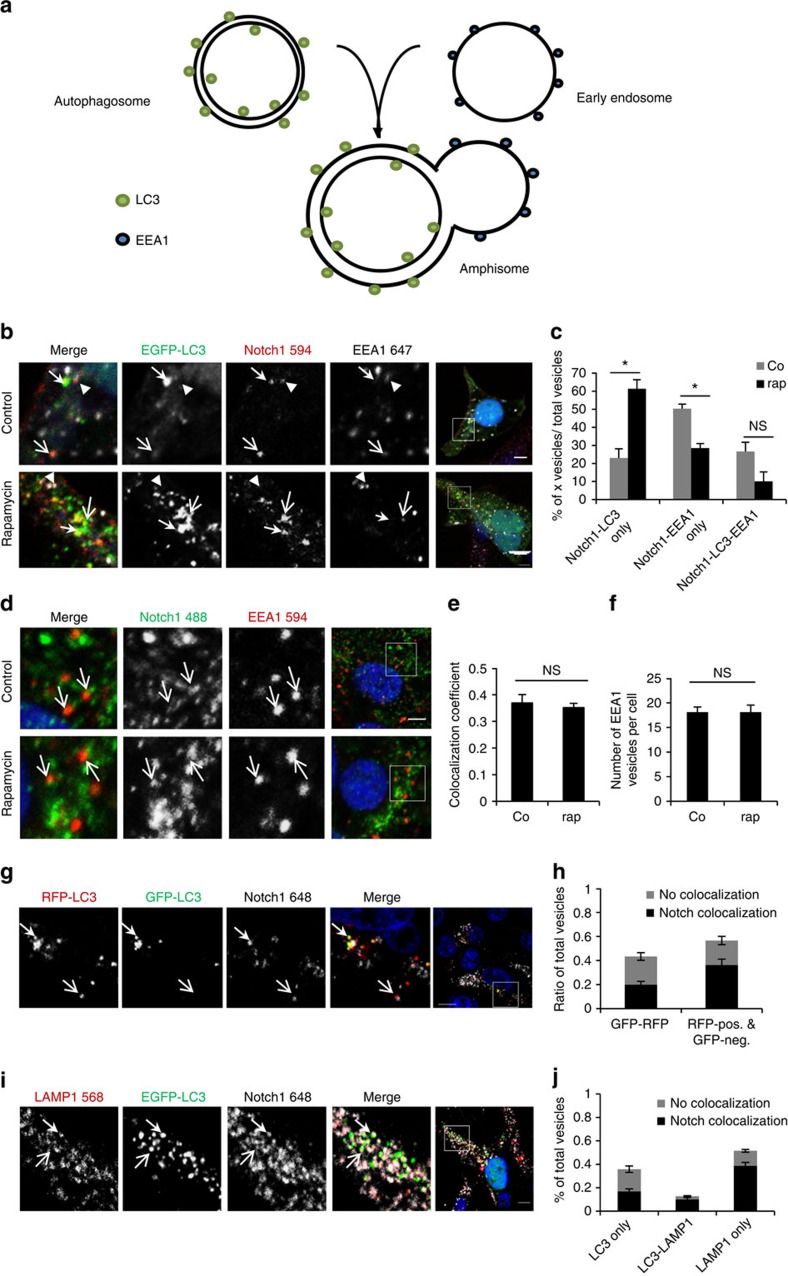
Notch1 can enter autophagosomes independently from endocytosis. (**a**) Autophagosomes (LC3-positive) can fuse with early endosomes (EEA1-positive) forming amphisomes. (**b**) HEK cells were transfected with pEGFP-LC3 and then treated with DMSO (control) or rapamycin, immunostained for Notch1 (red) and EEA1 (white) and DAP-stained (blue). ▸ shows Notch1-EEA1-only colocalization, → indicates LC3-Notch1-only colocalization, → indicates triple colocalization. Scale bar, 5 μm. (**c**) Quantification of vesicles positive for Notch1 and LC3, Notch1 and EEA1, or Notch1 and LC3 and EEA1, in DMSO (Co, grey) and rapamycin (rap, black) conditions. The percentage for each relative to total counted vesicles is shown. *P<0.05, unpaired *t-*test. *n*=3. Error bars=s.e.m. (**d**) HEK cells were treated with DMSO (control) or rapamycin for 8 h. Arrows show Notch1- and EEA1-positive vesicles. White box indicates location of enlargement shown in the three preceding images. Scale bar, 5 μm (**e**) Quantification of confocal images (Mander's colocalization) between Notch1 and EEA1 from 3d. NS denotes not significant, unpaired *t*-test. *n*=3. Error bars=s.e.m. (**f**) Quantification of number of total EEA1 vesicles in DMSO- and rapamycin-treated conditions. NS denotes not significant, unpaired *t*-test. *n*=3. Error bars=s.e.m. (**g**) HEK cells were transfected with mRFP-GFP-LC3 and stained for Notch1. → shows Notch1 colocalization with RFP- and GFP-positive structures. → shows Notch1 colocalization with RFP-positive, GFP-negative structures. Scale bar=5 μm. (**h**) Quantification of vesicles positive for GFP and RFP with/without Notch1, and RFP-only vesicles with/without Notch1. Total number of vesicles is total number of (GFP and RFP)+RFP-only positive vesicles. The number of Notch1-positive or -negative vesicles is divided by the total number of vesicles. *n*=3. Error bars=s.e.m. (**i**) HEK cells were transfected with pEGFP-LC3 and stained against LAMP1 and Notch1. → shows Notch1 colocalization with EGFP-LC3 and LAMP1. → shows Notch1 colocalization with LC3-positive, but LAMP1-negative structures. Scale bar, 5 μm. (**j**) Quantification of vesicles positive for LC3, or LC3-LAMP1 double-positive, or LAMP1- positive vesicles with/without Notch1. Total number of vesicles is the total number of LC3-only+LC3-LAMP+LAMP1-only positive vesicles. The number of Notch1-positive or -negative vesicles is divided by the total number of vesicles. *n*=3. Error bars=s.e.m.

As Notch1 is known to be degraded by endocytosis^14^, this suggested a possible route by which Notch1 could enter the autophagic pathway as, once formed, autophagosomes are able to fuse with endosomes forming amphisomes^20^, before finally fusing with lysosomes (Fig. 3a). Consistent with the canonical Notch pathway, Notch1 could be seen to localize to early endosomes (EEA1-positive vesicles), some of which were also positive for LC3, demonstrating an intersection of these two pathways (Fig. 3b,c, Supplementary Fig. 4a). Importantly, use of this triple staining allowed us to more accurately assess the localization of Notch1 in the autophagic and endocytic pathways. Notch1 was identified in not only EEA1-positive, LC3-negative early endosomes, and EEA1-positive, LC3-positive amphisomes, but also in LC3-positive, EEA1-negative autophagosomes (Fig. 3a–c, Supplementary Fig. 4a) suggesting Notch1 is also seen in autophagosomes that may have not fused with the endocytic pathway. Furthermore, on autophagy induction with rapamycin, only the colocalization of Notch1 with LC3-positive, EEA1-negative structures (autophagosomes) increased (Fig. 3b,c). Colocalization of Notch1 with EEA1-positive structures (either LC3-positive or -negative) was not enhanced by rapamycin treatment and total EEA1 vesicles number was not increased (Fig. 3d–f).

To further characterise the location of Notch1 in the autophagic pathway, we made use of tandem-tagged, mRed fluorescent protein-green fluorescent protein-LC3 (mRFP-GFP-LC3). This reporter is able to distinguish between autophagosomes and autolysosomes formed after the fusion of autophagosomes with lysosomes^21^, another potential intercept point for autophagy and the canonical Notch signalling pathway. The acidity of autolysosomes quenches GFP, so that autolysosomes are only RFP-positive, whereas autophagosomes are double-positive. Nearly 50% of the GFP–RFP-positive autophagosomes colocalized with Notch1, suggesting that Notch1 can also enter the autophagy pathway before autophagosome–lysosome fusion (Fig. 3g,h, Supplementary Fig. 4b,c). Likewise, immunostaining of cells for Notch1 and the lysosomal marker, LAMP1, in the presence of GFP–LC3 demonstrated that nearly half of the LC3-positive, LAMP1-negative vesicles (representing autophagosomes before fusion with lysosomes) colocalized with Notch1 (Fig. 3i,j, Supplementary Fig. 4d).

### Notch1 can enter autophagosomes in precursor structures

**Figure 4:**
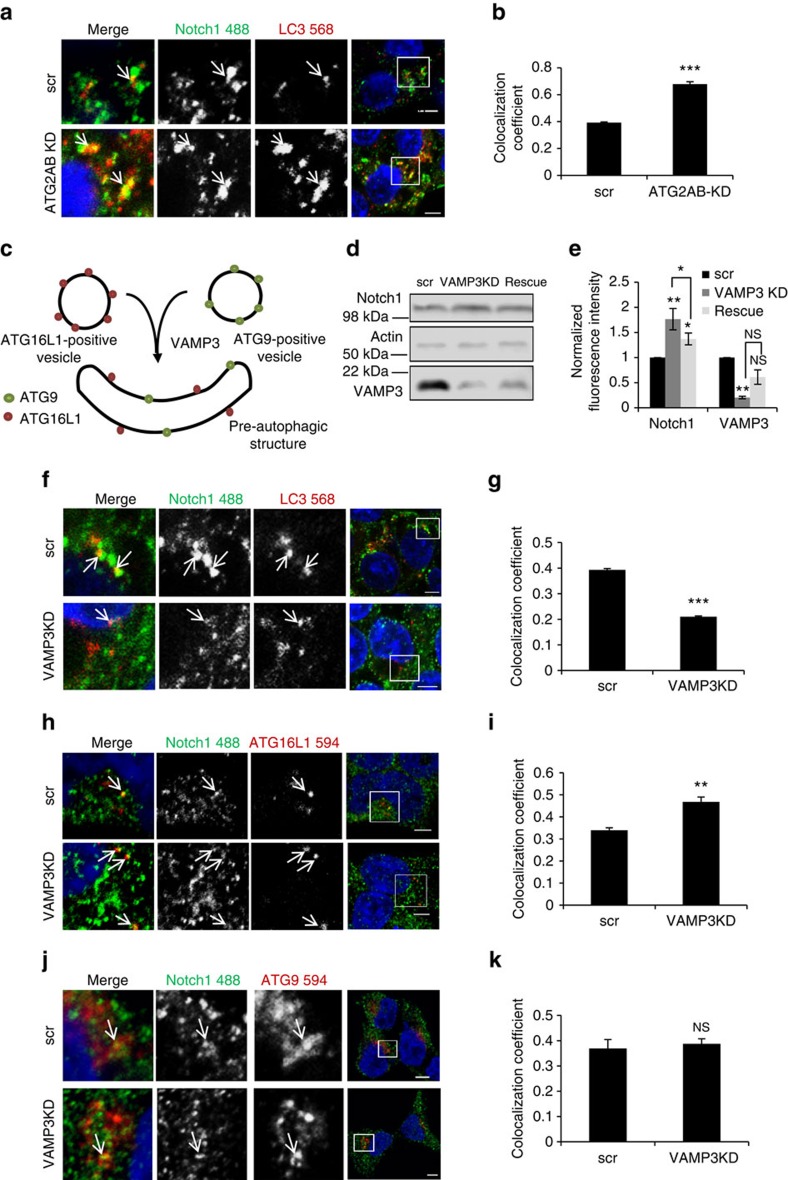
Notch1 can enter the autophagosomes in precursor structures. (**a**) Effect of inhibition of autophagy pathway components downstream (ATG2A and ATG2B) of LC3 on the colocalization of Notch1 with LC3. HEK cells were transfected with scrambled siRNA (scr) or ATG2AB siRNA. The arrows show LC3 and Notch1 colocalization. Scale bar, 5 μm. (**b**) Measurement of colocalization coefficient between Notch1 and LC3 in scrambled control and ATG2A/B knockdown conditions using Volocity software. ***P<0.001 by unpaired *t-*test relative to scrambled siRNA (scr) transfection. *n*=3. Error bars=s.e.m. (**c**) Schematic of VAMP3-mediated fusion of ATG16L1 and ATG9 vesicles forming ATG16L1- and ATG9-double-positive pre-autophagosomal structures. (**d**) Representative western: Protein levels of Notch1 and VAMP3 were assessed by western blot with actin loading control after VAMP3 knockdown ±VAMP3 cDNA rescue. (**e**) Quantification of western blots for Notch1 and VAMP3, relative to actin and normalized for the relevant control. *P<0.05 or *P<0.01 by paired *t-*test. NS denotes not significant. *n*=4. Error bars=s.e.m. (**f**) HEK cells were transfected with scrambled siRNA (scr) or VAMP3 siRNA. Arrows show LC3 and Notch1 colocalization. Scale bar, 5 μm (**g**) Colocalization coefficient between Notch1 and LC3 shown in f using Volocity software. ***P<0.001 by unpaired *t-*test relative to scrambled siRNA (scr) transfection. *n*=3. Error bars=s.e.m. (**h**) HEK cells were transfected with scrambled siRNA (scr) or VAMP3 siRNA. Arrows show ATG16L1 and Notch1 colocalization. Scale bar=5 μm. (**i**) Colocalization coefficient between Notch1 and ATG16L1 shown in h using Volocity software. **P<0.01 by unpaired *t*-test relative to scrambled siRNA (scr) transfection. *n*=3. Error bars=s.e.m. (**j**) HEK cells were transfected with scrambled siRNA (scr) or VAMP3 siRNA. Arrows show ATG9 and Notch1 colocalization. Scale bar, 5 μm. (**k**) Colocalization coefficient between Notch1 and ATG9 shown in j using Volocity software. NS denotes not significant by unpaired *t*-test relative to scrambled siRNA (scr) transfection. *n*=3. Error bars=s.e.m.

To confirm that Notch1 can be recruited into autophagosomes during their biogenesis before completion, we used ATG2A/B knockdown to prevent closure of autophagosomes and halt their final steps of formation^22^. We found that the number of Notch1-LC3 double-positive structures increased after ATG2A/B knockdown (Fig. 4a,b, Supplementary Fig. 5a). In addition, ATG2A/B knockdown increases Notch1 levels significantly and the elevated Notch1 level can be rescued by ATG2A overexpression in such knockdown cells (Supplementary Fig. 5b,c). This suggested the incorporation of Notch1 into early autophagic structures, as a decreased colocalization would be expected if the fusion of completed autophagosomes with the endocytic pathway was required for the colocalization of Notch1 with LC3. Thus, Notch1 can enter autophagosomes during biogenesis and not only through fusion of completed autophagosomes with Notch1-containing endosomes.

Our above data suggest that Notch1 can be taken up by autophagosomes at early stages, in addition to via amphisomes. Thus, we attempted to further characterise its route by investigating its colocalization with early markers of the autophagy pathway. Notch1 is located on the plasma membrane and since previous studies have identified a route from the plasma membrane to autophagosomes^23^, we therefore investigated whether Notch1 could also be trafficked via this pathway. The formation of autophagosomes depends on trafficking of ATG16L1 and mATG9 from different clathrin-coated pits on the plasma membrane in distinct vesicles that ultimately fuse in a VAMP3-dependent fashion^24^,^25^.

After internalization from the plasma membrane, ATG16L1-containing vesicles subsequently converge with mATG9-containing vesicles, which go on to form autophagosomes^24^. The R-SNARE that regulates mATG9–ATG16L1 vesicle fusion is VAMP3 (ref. 24; Fig. 4c). Knockdown of VAMP3 inhibited autophagy and resulted in an increase in Notch1 levels, but no increase in Notch ligand levels, as expected (Supplementary Fig. 5d–f). Overexpression of VAMP3 rescued the elevated Notch1 level in these knockdown cells (Fig. 4d,e). Importantly, the colocalization of Notch1 and LC3 was significantly decreased by VAMP3 knockdown (Fig. 4f,g, Supplementary Fig. 5g), while the colocalization of ATG16L1 and Notch1 was increased (Fig. 4h,i, Supplementary Fig. 5h), and the colocalization of mATG9 and Notch1 remained the same (Fig. 4j,k, Supplementary Fig. 6a). These results suggest that Notch can be taken up into early autophagic precursors by endocytosis in ATG16L1 precursor structures and not by ATG9 structures.

### Notch1 can enter ATG16L1 precursors from the plasma membrane

**Figure 5:**
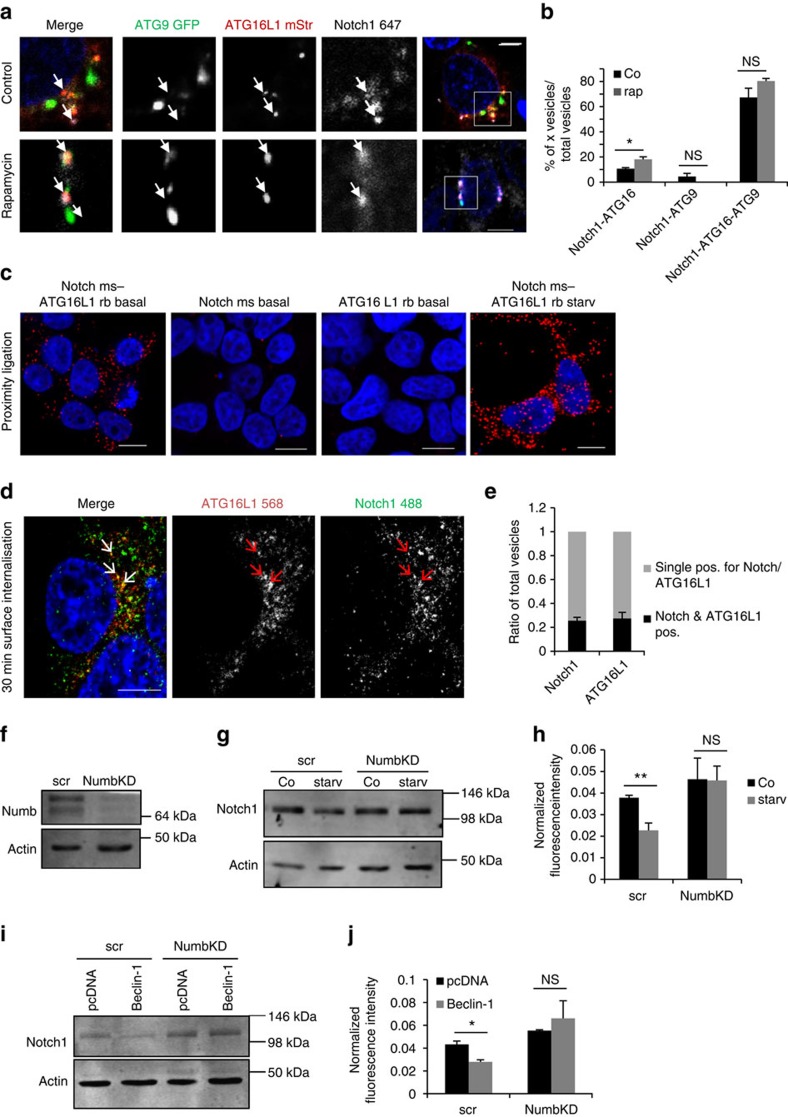
Notch1 can enter ATG16L1 precursors from the plasma membrane. (**a**) HEK cells were transfected with ATG9-pEGFP (ATG9 GFP) and pmStrawberry-ATG16L1 (ATG16L1 mStr), then treated with DMSO/rapamycin for 8 h. → shows ATG16L1-Notch1-only colocalization. Scale bar, 5 μm. (**b**) Quantification of vesicles positive for Notch1 and ATG16L1 but not ATG9, Notch1 and ATG9 but not ATG16L1, or Notch1 and ATG16L1 and ATG9 in DMSO (Co) and rapamycin (rap) conditions. The percentage positive/total counted vesicles is shown. * denotes *P*< 0.05 by unpaired *t*-test. *n*=3. Error bars=s.e.m. (**c**) HEK cells were starved for 2 h in HBSS or left in basal conditions and proximity ligation was performed using endogenous ATG16L1 and ms Notch1 antibodies. Scale bar, 10 μm. (**d**) Colocalization of Notch1 and ATG16L1 after surface internalization for 30 min. HEK cells were incubated with Notch1 antibody on ice and then for 30 min at 37 °C to allow internalization of labelled Notch1 cell surface receptors. Arrows indicate Notch1- (Alexa 488) and ATG16L1- (Alexa 568) double-positive structures. Scale bar, 5 μm. (**e**) Quantification of total Notch1-positive vesicles colocalizing with ATG16L1 and total number of ATG16L1-positive vesicles colocalizing with Notch1. *n*=3. Error bars=s.e.m. (**f**) HEK cells were transfected with Numb siRNA and western blotted for Numb and actin. (**g**) Western blot showing effect on Notch1 level following starvation in control and Numb knockdown conditions. HEK cells were transfected with Numb siRNA and scrambled siRNA and subsequently starved (2 h HBSS). (**h**) Quantification of blots from 5 g. Notch1 levels were compared between untreated (Co) and HBSS treated (starv) following Numb KD/scrambled siRNA. NS denotes not significant and **P<0.01 by unpaired *t*-test. *n*=3. Error bars=s.e.m. (**i**) Representative western blot showing the effect of Numb knockdown on Notch1 level after Beclin-1 overexpression. HEK cells were transfected with Numb siRNA or scrambled siRNA (scr) and subsequently transfected with pcDNA/Beclin-Flag. (**j**) Quantification of western blots from 5i. The effect of pcDNA or Beclin-1 overexpression on Notch1 levels is shown in control cells (scrambled) or following knockdown of Numb (Numb KD). *P<0.5 by unpaired *t*-test. *n*=3. Error bars=s.e.m.

To further test this hypothesis, we used triple staining of Notch1 with mATG9 and ATG16L1 to allow us to identify which of these precursor vesicles contained Notch1 (Fig. 5a and Supplementary Fig. 6b). Notch1 could be seen in mATG9-positive, ATG16L1-positive pre-autophagosome structures, in ATG16L1-positive, mATG9-negative vesicles and to a lesser extent in mATG9-positive, ATG16L1-negative vesicles. Since mATG9 traffics via early endosomes, known to contain Notch1, it is possible that these Notch1-positive, mATG9-positive, ATG16L1-negative vesicles may represent early endosomes. On autophagy induction with rapamycin, the colocalization of Notch1 with ATG16L1-only-positive vesicles, but not mATG9-only-positive vesicles was significantly increased (Fig. 5a,b). To further confirm the colocalization of Notch1 with ATG16L1, we performed proximity ligation assays. A clear signal indicating colocalization of Notch1 and ATG16L1 was seen under basal conditions, and this signal was strongly enhanced when autophagy was induced by starvation (Fig. 5c). The adaptor required for the internalization of ATG16L1 via clathrin-coated pits is AP2 (ref. 23). We were able to observe Notch1 localizing in ATG16L1/AP2 double-positive structures (Supplementary Fig. 6c), consistent with the idea that Notch1 can be internalized and trafficked to autophagosomes alongside ATG16L1. Notch1- and ATG16L1-positive but LC3-negative vesicles were observed, indicating that Notch1 can enter ATG16L1 vesicles before autophagolysosome formation (Supplementary Fig. 6d).

To further test whether Notch1 entered early autophagic structures directly from the cell surface, we labelled plasma membrane Notch1 using an anti-Notch1 antibody that recognizes its extracellular domain. HEK cells were incubated with the antibody on ice for 15 min, and then the temperature was raised to 37 °C to allow internalization of the labelled pool of plasma membrane Notch1. We detected colocalization of Notch1 and endogenous ATG16L1, indicating Notch1 uptake from the cell exterior into cytoplasmic ATG16L1 vesicles (Fig. 5d, Supplementary Fig. 7a). A quarter of Notch1-positive vesicles colocalized with ATG16L1 (Fig. 5e). Note that only a small proportion of the cell surface-labelled Notch1 is in these ATG16L1 vesicles, which would be expected since only some of these Notch1 receptors will be internalized via this pathway, or be within ATG16L1-positive vesicles at this timepoint. The negative control, with Alexa 488 secondary antibody only, did not show labelling (Supplementary Fig. 7b). Likewise, no ATG16L1–Notch1 colocalization was seen in cells when the Notch1 was not allowed to internalise (Supplementary Fig. 7b).

To identify the mechanism by which Notch1 may be targeted to autophagosomes, we looked at the effect of Numb, a known adaptor for Notch1 degradation through endocytosis^26^. siRNA knockdown of Numb was able to ablate the effect of starvation and Beclin-1 overexpression on Notch1 protein levels (Fig. 5f–j). This suggests that Numb also acts upstream of the autophagy pathway (see schematic diagram of Supplementary Fig. 7c) and may be an adaptor for both autophagy and endocytosis.

Overall, our data suggest that Notch1 is degraded by autophagy and that it is trafficked to autophagosomes from the plasma membrane in ATG16L1-containing vesicles, as well as entering the autophagic pathway via its endocytic route.

### Notch1 in primary neurons is affected by autophagy

**Figure 6:**
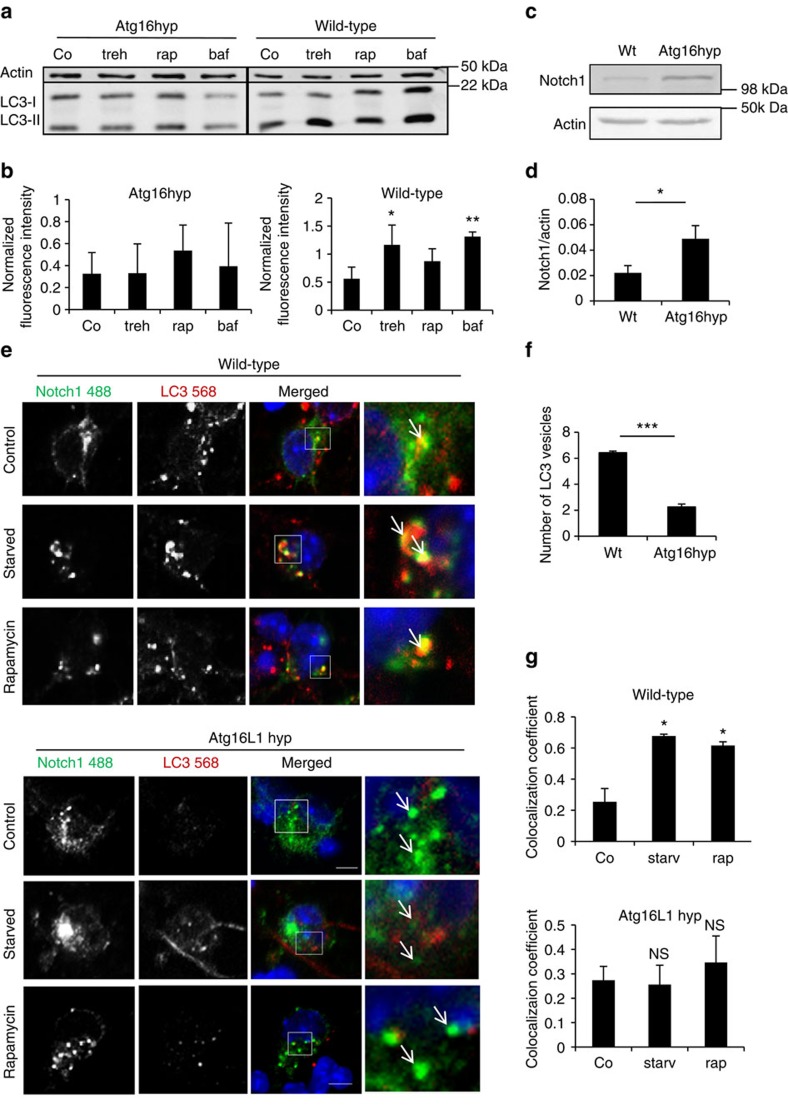
Autophagy modulates Notch1 in primary neurons. (**a**) Representative western blot of LC3-II levels in wild-type and Atg16L1 hypomorph (Atg16hyp) mice. E15.5 wild-type and Atg16L1 hyp primary cortical neurons were lysed after treatment with DMSO (Co), trehalose (treh), rapamycin (rap) and bafilomycin A1 (baf) for 8 h and analysed by western blot. (**b**) LC3-II protein level relative to actin loading control from western blots in a. *P<0.05 and **P<0.01 by unpaired *t*-test. *n*=3. Error bars=s.e.m. (**c**) Representative western blot showing Notch1 level in brain lysates from wild-type and Atg16L1 hyp 5-week-old animals. (**d**) Quantification of western blots for Notch1 in wild-type and Atg16L1 hypomorph mice (Atg16 hyp). *P<0.05 by unpaired *t*-test. *n*=4 mice per group. Error bars=s.e.m. (**e**) Effect of pharmacological autophagy modulation on the colocalization of Notch1 with LC3 in wild-type (E15.5) and Atg16L1 hyp primary cortical neurons. Neurons were treated with DMSO (Control), starved with HBSS for 2 h or treated with rapamycin (8 h). Arrows denote Notch1 and LC3 colocalization. Scale bar, 5 μm. (**f**) Number of LC3 vesicles in neurons from wild-type compared to Atg16L1 hypomorph (Atg16hyp) mice. Mean number of LC3 vesicles per cell is shown based on counting vesicles in at least 15 cells per condition ***P<0.001 by unpaired *t*-test. *n*=3. Error bars=s.e.m. (**g**) Mander's colocalization coefficient of Notch1 and LC3 in wild-type and Atg16L1 hyp primary neurons. *P<0.05 or ***P<0.001 by unpaired *t*-test relative to control (Co). Error bars represent standard error of the mean. *n*=3. Error bars=s.e.m.

To test the consequences of the regulation of Notch signalling by autophagy, we used a mouse model with a hypomorphic mutation in *Atg16L1*. This mouse has modestly impaired autophagy, and is therefore more likely to have physiological relevance compared with autophagy knock-outs^27^. While autophagic stimulation with trehalose^28^, and lysosomal inhibition with bafilomycin increased LC3-II levels in primary neurons from wild-type mice, these effects were attenuated in neurons from Atg16L1 hypomorph animals (Fig. 6a,b). Consistent with the previous data, the ATG16L1 level was reduced in the brain lysates of the Atg16L1 hyp mice compared with wild-type mice (Supplementary Fig. 8a), the protein levels of Notch1, NICD and Hes1 were elevated in the brain lysates of Atg16L1 hypomorphs, compared with wild-type mice (Fig. 6c,d, Supplementary Fig. 8b). In the Atg16L1 hypomorph mice, the numbers of LC3 vesicles, assessed by immunostaining, were 1/3 of wild-type levels (Fig. 6e,f, Supplementary Fig. 8c). In wild-type primary neurons, Notch1–LC3 colocalization was increased after starvation or rapamycin treatment, whereas no obvious Notch1–LC3 colocalization was observed in Atg16L1 hypomorph primary neurons (Fig. 6g) and the colocalization did not increase after starvation or rapamycin treatment (Fig. 6g).

### Increased Notch1 is linked to elevated stem cell staining

**Figure 7:**
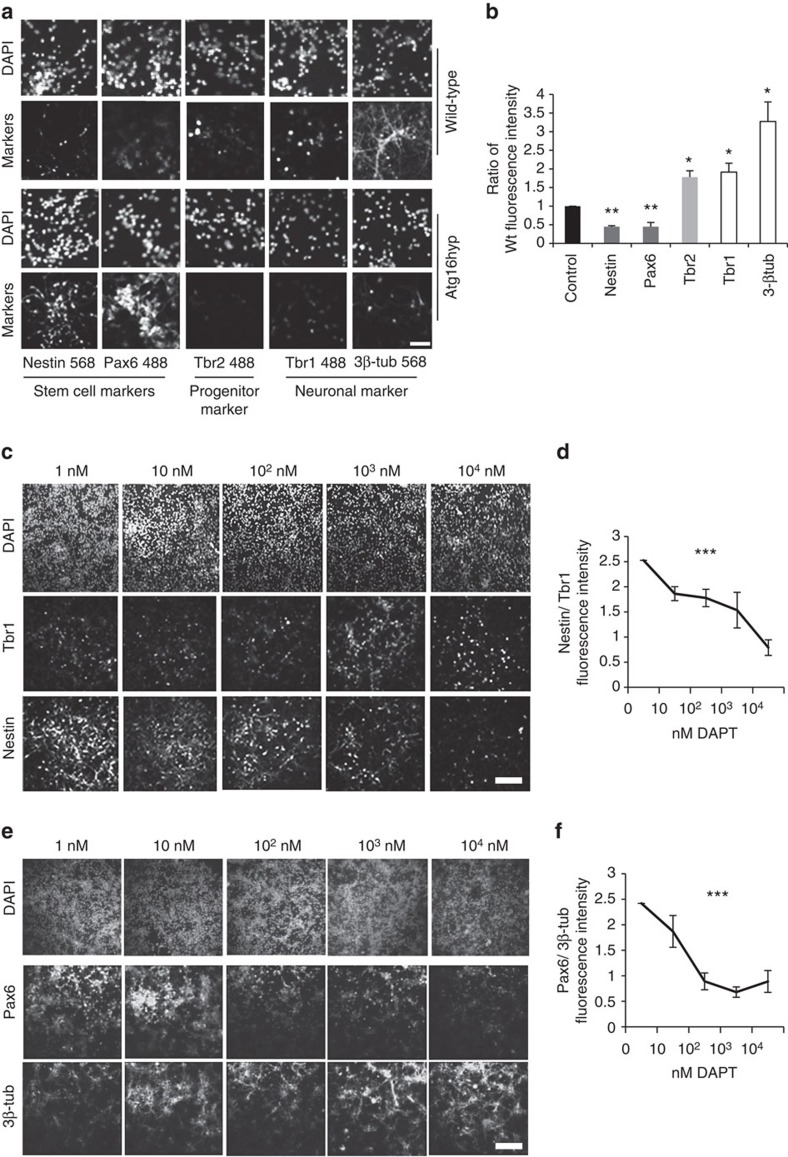
Increased Notch1 in Atg16L1 hyp increases stem cell staining. (**a**) Effect of decreased Atg16L1 on differentiation of primary neurons in culture. Wild-type and Atg16L1 hyp primary cortical neurons were stained for stem cell markers (Nestin, Pax6), progenitor marker Tbr2 and neuronal markers (Tbr1, β3-tub). Scale bar=100 μm is valid for all panels. (**b**) Quantification of relative fluorescence intensity of immunostaining for markers of stem cells (dark grey bars), progenitors (light grey bars) neurons (white bars) as shown in a. Intensity is expressed as a ratio of wild-type (Wt) to Atg16L1 hypomorph (Atg16hyp) *P<0.05 by paired *t*-test. *n*=3. Error bars=s.e.m. (**c**) Effect of Notch pathway inhibition (by DAPT) on the differentiation of Atg16L1 hypomorph primary neurons. Neurons from E15.5 Atg16L1 hypomorph (Atg16hyp) mice were treated with increasing concentration of DAPT and stained for Nestin and Tbr1. Scale bar, 200 μm is valid for all panels. (**d**) Quantification of fluorescence intensity of immunostaining for stem cell marker Nestin and neuronal marker Tbr1 in neuronal cultures from Atg16L1 hypomorph mice following DAPT treatment at different concentrations as shown in c. The values are shown as a ratio of stem cell marker relative to neuronal marker to correct for any differences in cell number. ***P<0.001 by analysis of variance. *n*=3. Error bars=s.e.m. (**e**) Effect of Notch pathway inhibition (by DAPT) on the differentiation of Atg16L1 hypomorph primary neurons. Neurons from E15.5 Atg16L1 hypomorph (Atg16hyp) mice were treated with increasing concentration of DAPT and stained Pax6 and 3β-tub. Scale bar, 200 μm is valid for all panels. (**f**) Quantification of stem cell marker Pax6 relative to neuronal marker 3β-tub as described in e. *n*=3. Error bars=s.e.m.

Since Notch1 is crucial in development and stem cell maintenance, we assessed stem cell (Nestin, Pax6), progenitor (Tbr2) and neuronal markers (Tbr1, 3β-tubulin) in wild-type and Atg16L1 hypomorph primary neurons^29^. Wild-type neurons had half the fluorescence intensity of Nestin and Pax6 staining compared with the Atg16L1 hypomorph neurons, while the Tbr1, Tbr2 and 3β-tub intensities were at least double in wild-type versus hypomorph mice (Fig. 7a,b). The level of the markers were tested by western blots and the level of Nestin and Pax6 were significantly higher in the Atg16L1 hypomorph mice compared with wild-type, whereas the level of the neuronal markers Tbr1 and 3β-tubulin were significantly lower (Supplementary Fig. 9a,b). Therefore, Atg16L1 hypomorph mice had a significantly higher proportion of stem cells in neuronal cultures compared with wild-type. To see whether this effect was Notch-related, we applied the γ-secretase inhibitor DAPT^30^, which blocks the cleavage of Notch1 to NICD, and thus blocks Notch signalling. The efficiency of DAPT can be assessed by the level of NICD, which declined as DAPT concentrations were increased (Supplementary Fig. 9c, d). Atg16L1 hypomorph primary neurons showed an increase in the intensities of Tbr1 and 3β-tub and a decrease in the intensities of Nestin and Pax6 with increasing concentrations of DAPT (Fig. 7c–f). In concert, increased concentrations of DAPT also caused a significant decline of Nestin/Tbr1 and Pax6/3β-tub relative staining intensities in the same cells (Fig. 7c–f), indicating that inhibition of Notch signalling causes a reversion of the stem cell phenotype and pushes cells to a more differentiated state, which is more similar to the wild-type. The same trend was visible in wild-type cells, when DAPT was applied (Supplementary Fig. 9e–h). Western Blot analysis also showed significant decrease of Nestin and Pax6 levels and increase of Tbr1 and 3β-tubulin levels after DAPT treatment in Atg16L1 hypomorph and wild-type primary neurons (Supplementary Fig. 10). Since the impaired differentiation of stem cells towards neurons in the Atg16L1 hypomorph neurons can be reversed by impeding Notch signalling, these data suggest that this stem cell phenotype is Notch related. However, we cannot exclude contributions from other putative pathways.

### Atg16L1 hypomorph mice have developmental retention

**Figure 8:**
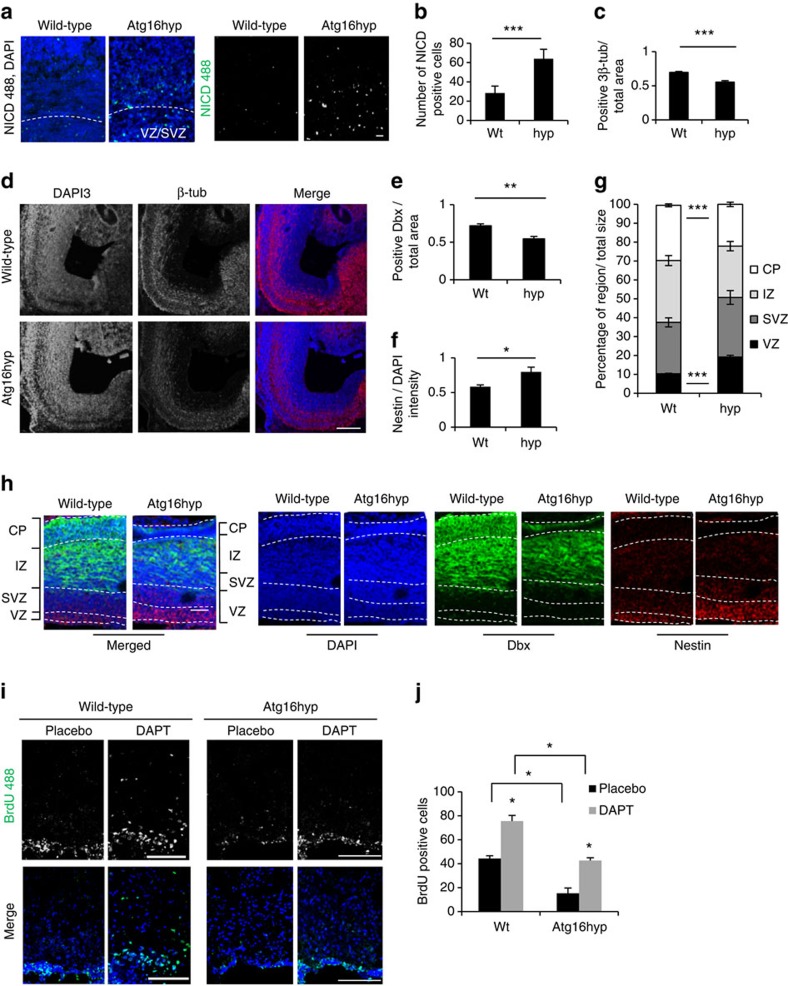
Atg16L1 hyp mice show a delay in development in the brain. (**a**) NICD immunostaining (green) in E15.5 brain slices from wild-type and Atg16L1 hypomorph (Atg16hyp) embryos counterstained with DAPI (blue). The ventricular zone (VZ) and subventricular zone (SVZ) is marked with dotted line. Scale bar, 10 μm. (**b**) Quantification of the number of NICD positive cells in wild-type (Wt) and Atg16L1 hypomorph (hyp) brain slices. ***P<0.001 by unpaired *t*-test. *n*=3. Error bars=s.e.m. (**c**) Quantification of the neuronal area in wild-type (Wt) and Atg16L1 hypomorph (hyp) brain slices. The 3β-tub area was divided by the DAPI-positive area to normalize for total brain size. ***P<0.001 by unpaired *t*-test. *n*=6. Error bars=s.e.m. (**d**) Effect of decreased levels of Atg16L1 on brain development. E15.5 wild-type or Atg16L1 hypomorph (Atg16hyp) brains immunostained for 3β-tub. Scale bar, 50 μm. (**e**) Quantification of the neuronal area in wild-type (Wt) and Atg16L1 hypomorph (hyp) brain slices. The Dbx-positive area was divided by the DAPI-positive area to normalize for total brain size. **P<0.01 by unpaired *t*-test. *n*=6. Error bars=s.e.m. (**f**) Quantification of the stem cell marker immunostaining intensity in wild-type (Wt) and Atg16L1 hypomorph (hyp) brain slices. The Nestin intensity was divided by the DAPI intensity to normalize for total cell number. *P<0.05 by unpaired *t*-test. *n*=3. Error bars=s.e.m. (**g**) Quantification of the region size of the ventricular zone (VZ), subventricular zone (SVZ), intermediate zone (IZ) and the cortical plate (CP) in wild-type and Atg16L1 hypomorph mice. The size of each region was normalized to the total size of all regions. ***P<0.001 by unpaired *t*-test. *n*=3. Error bars=s.e.m. (**h**) Nestin and Dbx immunostaining in E15.5 wild-type and Atg16L1 hypomorph mice brains marked with dotted lines for ventricular zone (VZ), subventricular zone (SVZ), intermediate zone (IZ) and the cortical plate (CP). Scale bar, 50 μm. (**i**) BrdU and DAPI immunostaining in 9–11 months wild-type and Atg16L1 hypomorph mouse brains. Scale bar, 100 μm. (**j**) Quantification of BrdU-positive cells in wild-type (Wt) and Atg16L1 hypomorph (Atg16hyp) brain slices. *P<0.05 by unpaired *t*-test. *n*=3. Error bars=s.e.m.

Next, we confirmed the stem cell phenotypes in different tissues from mice. We observed increased NICD staining in Atg16L1 hypomorph brains compared with wild-type (Fig. 8a). The total number of NICD-positive cells is more than double in the Atg16hyp mice compared with wild-type (Fig. 8b). Stem cells are mainly situated around the ventricles, in the ventricular zone, and migrate to the outer zone to develop into neurons. The bigger the neuronal zones are, the more differentiated a brain is^31^. When E15.5 embryonic brain slices were stained with differentiation markers, Atg16L1 hypomorph mice showed 1/3 smaller 3β-tub- and Dbx- (neuronal marker)^32^ positive regions around the ventricles (Fig. 8c–e and Supplementary Fig. 11a). The intensity of Nestin (stem cell marker) staining was stronger in Atg16L1 hypomorph brain slices, compared with wild-type (Fig. 8f). A direct comparison of the brains of the Atg16L1 hypomorph mice and wild-type mice shows significant changes in the size of the ventricular zone and the cortical plate (Fig. 8g,h). The region of stem cells, ventricular zone, is double the size in the Atg16L1 hypomorph mice compared with the wild-type. Consistent with our previous data, the cortical plate, the region of mature neurons, is significantly smaller in the Atg16L1 hypomorph compared with wild-type mice (Fig. 8g,h).

Previous studies in adult mice brains have shown that activation of Notch1 signalling inhibits proliferation and differentiation, and that Notch1 inhibition increases proliferation^33^,^34^. Thus, a prediction of our data is that the Notch1 hyperactivity and impaired differentiation in the adult hypomorph mice should decrease neurogenesis. Adult wild-type and Atg16L1 hypomorph mice (9–11 months of age) were injected with BrdU for 6 days to label proliferating cells. The number of BrdU-positive cells was significantly higher in wild-type compared with Atg16L1 hypomorph mice (Fig. 8i,j). Treatment with the Notch inhibitor DAPT for 6 days before BrdU injection significantly increased the BrdU-positive cells in both wild-type and Atg16L1 hypomorph mice (Fig. 8i,j). Consistent with our previous results, Notch1 levels were significantly higher in the Atg16L1 hypomorph brains compared with wild-type (Supplementary Fig. 11b,c). Thus, these data suggest that the increased Notch1 resulting from defective autophagy is impairing differentiation, which is known to be associated with decreased neurogenesis and cell proliferation in adult mice^33^,^34^. Furthermore, this cell division phenotype can be ameliorated by DAPT, suggested that it is Notch related.

### Delayed bone marrow/gut differentiation in Atg16L1 hypomorphs

**Figure 9:**
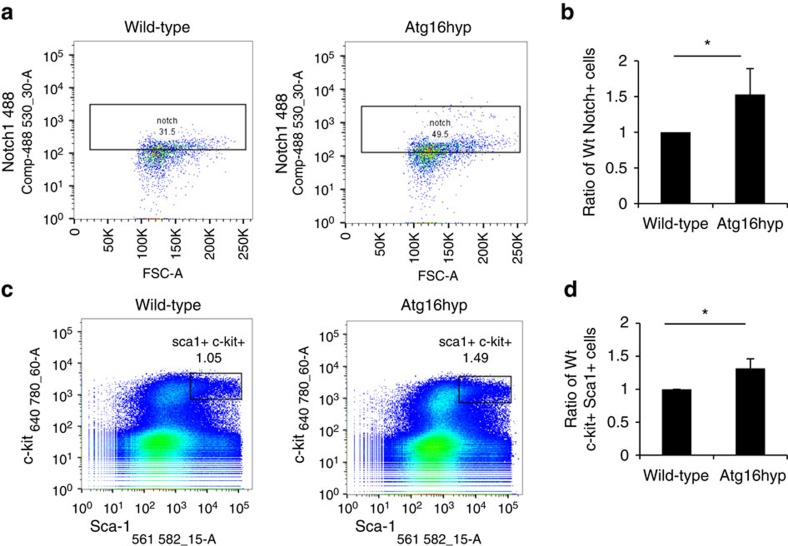
Delayed bone marrow/gut differentiation in Atg16L1 hypomorphs. (**a**) Dot plots showing representative FACS analysis of Notch1 level in the stem cell population of bone marrow cells isolated from 6 week old wild-type and Atg16hyp mice. Stem cell population was gated and defined as 7AAD-negative, lineage-negative, c-kit-positive and Sca1-positive and Notch1-positive cells. Percentage of stem cells positive for Notch1 above an arbitrary threshold value is shown. (**b**) The per cent of cells staining positive for Notch1 (as described in **a**). The graph shows the ratio of Notch1-positive cells gated in Atg16L1 hypomorph (Atg16hyp) to wild-type (Wt) gated cells. *P<0.5 by paired *t*-test. *n*=6. Error bars=s.e.m. (**c**) Effect of decreased ATG16L1 levels on differentiation of bone marrow stem cells. Representative dot plots from FACS analysis of bone marrow cells from 6 weeks old wild-type and Atg16L1 hypomorph (Atg16hyp) mice. The black box indicates the gated population identified as stem cells, defined as 7AAD-negative, lineage-negative, c-kit-positive and Sca1-positive cells. (**d**) Quantification of gated population of stem cells as illustrated in c. The ratio of Atg16L1 hypomorph (Atg16hyp) gated cells to wild-type (Wt) gated cells is shown. *P<0.5 by paired *t*-test. *n*=9. Error bars=s.e.m.

**Figure 10:**
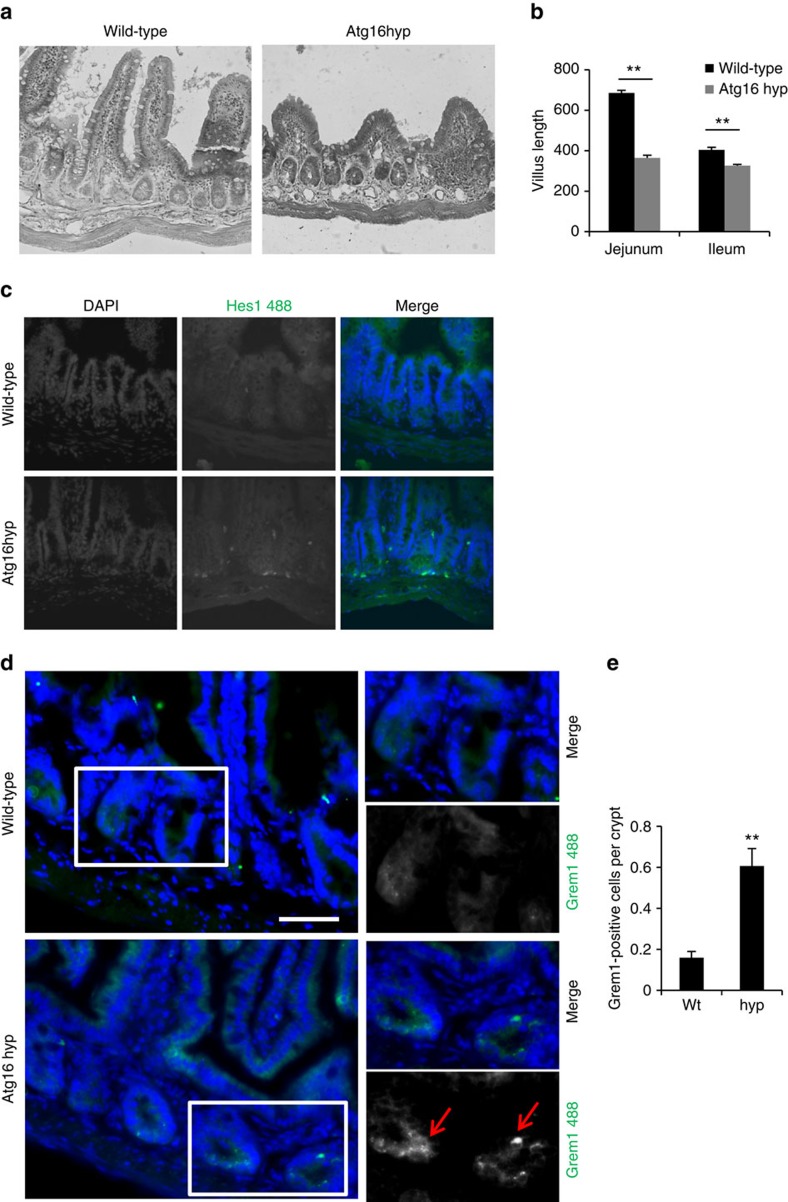
Atg16L1 hypomorphs show developmental retention in the gut. (**a**) Effect of decreased Atg16L1 levels on growth of gut villi length. Representative gut section from wild-type and Atg16L1 hypomorph (Atg16hyp) 10–15 weeks old mice. (**b**) Quantification of villi length from wild-type and Atg16L1 hypomorph (Atg16hyp) 10–15 weeks old mice. **P<0.01 by unpaired *t*-test. *n*=4. Error bars=s.e.m. (**c**) Hes1 immunostaining in gut from wild-type and Atg16L1 hypomorph (Atg16hyp) mice. (**d**) Grem1 immunostaining in gut from wild-type and Atg16L1 hypomorph (Atg16hyp) mice. (**e**) Quantification of Grem1-positive cells per crypt in wild-type and Atg16L1 hypomorph (Atg16hyp) mice. **P<0.01 by paired *t*-test. *n*=3. Error bars=s.e.m. Scale bar, 50 μm.

Since Notch1 is widely expressed in many tissues, we also analysed the bone marrow of the mice. Stem cell and progenitor cells of the bone marrow are characterized by low expression of lineage markers and they are positive for Sca1 and c-kit. The heterogenous population of lineage-negative (linlow) cells (marks not fully differentiated cells) show significantly higher Notch1 staining in the Atg16L1 hypomorph in comparison with the wild-type (Fig. 9a,b). In the bone marrow, we similarly observed an increase of linlow/− Sca1+ and c-kit+ double-positive cells (containing hematopoietic stem cells) in the Atg16L1 hypomorph, compared with wild-type mice, indicating that Atg16L1 hypomorph bone marrow cells contain a higher number of less differentiated cells (Fig. 9c,d). The final tissue we analysed was the gut. ATG16L1 is a risk allele for Crohn's disease^27^ and Notch signalling in the intestinal stem cell population is essential for maintenance of the normal architecture of the gut epithelium^35^. We, therefore, performed histological examination of the ileum and jejunum. Transverse sections through these two regions of the small intestine showed a striking reduction in villus length in Atg16L1 hypomorph mice, compared with wild-type siblings, consistent with impaired stem cell differentiation (Fig. 10a,b). This was associated with an increase in Hes1 antibody staining in the crypt/stem cell region of Atg16L1 hypomorph mice, suggesting that altered Notch signalling may underlie the observed structural changes (Fig. 10c). In addition to Hes1, a known gut stem cell marker, we tested another gut stem cell marker Gremlin 1 (Grem1). There were significantly more Grem1-positive cells observed in the Atg16L1 hypomorph jejunum crypts compared with the wild-type (Fig. 10d,e), indicating that Notch also affects stem cells development in the gut.

## Discussion

Our study argues that autophagy regulates Notch signalling because it is one of the degradation routes for the receptor Notch1, which perturbation accounts for significant changes in the signalling output. These mammalian data contrast with two previous studies in Drosophila. The first suggested that *Drosophila ultraviolet-resistance-associated* gene (*UVRAG*), which is involved in both endocytosis and autophagy, regulates organ rotation, by influencing endocytic Notch degradation and rules out autophagy explicitly, since a number of ATG-deficient mutants including ATG1, 6 and 7, do not display the same loss-of-function phenotype^36^. The second study reported that the Notch pathway is hyperactivated in *Drosophila* autophagy mutant somatic follicle cells and, while no mechanism was defined, the authors suggested that this may have been due to altered Notch ligand levels^37^. Our findings did not confirm this hypothesis, since we showed that autophagy up- or downregulation did not affect Notch ligand levels.

We demonstrate a developmental retention in neurogenesis, haematopoiesis and in the gut villi in Atg16L1 hypomorph mice, all developmental phenomena, which are dependent on Notch1 (refs 33, 38). Our data argue that this is likely due to impaired autophagic degradation of Notch1 and thus hyperactivated Notch signalling, as these mice have elevated levels of Notch1, NICD and Hes1. Importantly, the impaired differentiation of the Atg16L1 hypomorphic neurons was rescued by a Notch inhibitor, suggesting that these effects are Notch related.

These alterations in stem cell differentiation are likely to be generic effects of autophagy impairment in the Atg16L1 hypomorph mice, and not specific autophagy-independent consequences of the ATG16L1 activity, since the effects of ATG16L1 depletion of Notch and its signalling were mimicked by ATG7 siRNA in cell culture, and the reverse effects were seen when autophagy was upregulated by overexpressing Beclin-1, or by rapamycin treatment or starvation. Futhermore, the impaired stem cell differentiation phenotypes are unlikely to be artefactual consequences of changes of marker expression, as we used five markers in the neurons—stem cell (Nestin, Pax6), progenitor (Tbr2) and neuronal markers (Tbr1, 3β-tubulin)—distinct markers in the bone marrow and morphological assessments in the gut. Furthermore, these are predictable consequences of Notch hyperactivation^33^,^35^,^39^ and are seen in the context of increased Notch activity in these tissues.

Our approach contrasts with previous mammalian studies that have used autophagy null mice to demonstrate developmental abnormalities, which have been attributed to overall impairments of nutrient recycling^40^,^41^, as opposed to altered levels of a specific autophagy substrate, as we have shown here. Our findings are based on *in vivo* and primary neuron data in an autophagy hypomorph model as opposed to a null mutant, and suggest that modest alterations of autophagy are sufficient to impact on Notch signalling and its downstream effects on stem cell differentiation, a conclusion that would not be possible with complete null models. Furthermore, it is possible that complete loss of autophagy may result in different phenotypes compared with partial impairment. For example, complete loss of the *FIP200* gene that regulates autophagy leads to apoptosis and consequent depletion of the neuronal stem cell pool^42^. Thus, complete loss of autophagy genes may not cause the same phenotypes as we have observed in the hypomorph mouse. Indeed, we would argue that studies in a hypomorphic context have greater physiological relevance, since the impairments of autophagy in diseases like forms of neurodegeneration and Crohn's disease are not complete.

## Methods

### Constructs

pmStrawberry–ATG16L1 and the Beclin-Flag was described in Cadwell *et al.*^27^ and Luo *et al.*^43^, respectively. pCMV-hATG7wt was a kind gift from Dr Isei Tanida. siRNA-resistant ATG2A–GFP was a kind gift from Dr. Noboro Mizushima and VAMP3-HA was a kind gift from Dr Andrew Peden. ATG9L1–pEGFP was a kind gift from Dr Yoshinori Takahashi and pEGFP–LC3 and mRFP–GFP–LC3 were kind gifts from Tamotsu Yoshimori.

### Cell culture

HEK cells were cultured in Dulbecco's Modified Eagle's Medium (Life Technologies) containing 4,500 mg l^−1^ glucose (Sigma-Adrich) and supplemented with 10% FBS (Sigma-Adrich), 100 units per ml penicillin (Sigma-Adrich), 100 μg ml^−1^ streptomycin (Sigma-Adrich), and 2 mM L-glutamine (Life Technologies) at 37 °C and 5% carbon dioxide.

### Transient transfection

HEK cells were plated to be 70% confluent and transfected the following day. For one six-well plate, 2 μg (Beclin-Flag, pcDNA) or 0.5 μg (pmStrawberry–ATG16L1, ATG9L1–pEGFP, pEGFP–LC3, mRFP–GFP–LC3) DNA transfected with 7.5 μl TransIT-2020 (Mirus) following the manufacturer's protocol.

### siRNA knockdown and rescue

HEK cells were plated into six-well plates to reach 80% confluency. For each well, 4 μl of 20 μM On-TARGETplus SMARTpool siRNA (ATG16L1, ATG7 Dharmacon) or deconvoluted oligos (ATG16L1, ATG7 Dharmacon) or individual-specifc siRNA (see below, Thermo Scientific) was transfected with 5 μl Lipofectamine 2000 (Life Technologies) according to the manufacturer's protocol. siRNA transfection was repeated the following day. Cells were then incubated for 24 h.

For rescue, 0.5 μg of pmStrawberry-ATG16L1, pCMV-hATG7wt, siRNA resistant ATG2A-GFP and VAMP3-HA were transfected on day 3 with 7.5 μl TransIT-2020 (Mirus) following the manufacturer's protocol.

*siRNA sequence*. VAMP3 5′-GGCAGGCGCUUCUCAAUUU-3′

Atg2A 5′-GCAUUCCCAGUUGUUGGAGUUCCUA-3′

Atg2B 5′-AGGUCUCUCUUGUCUGGCAUCUUUA-3′

### Luciferase assay

HEK cells were transfected with a solution (SabioSciences) containing Notch firefly reporter and constitutively active Renilla contruct (3 μl per plate). After 48 h incubation, the Dual-Luciferase assay (Promega) was carried out following the manufacturer's protocol. The firefly luciferase signal is divided by Renilla luciferase signal to normalize for cell number.

### Immunoblotting

Samples were run by SDS-PAGE and transferred to low autofluorescence polyvinylidene difluoride membrane (Immobilon, FL, Millipore). Primary antibodies were used at the concentration described below overnight at 4 °C in blocking solution (1% milk powder in PBS). Secondary antibody (IR-Dye conjugation goat anti-mouse or -rabbit LI-COR Biosciences) was used at 1:50,000 dilution in blocking solution for 1 h. The membrane was scanned on the LI-COR Odyssey (LI-COR Biosciences) using the Image Studio software.

*Dilution of primary antibodies*. 1:250: Notch1 (ab52627, Abcam), NICD (ab8925, Abcam), Hes1(ab71559, Abcam), Dll1 (ab76655, Abcam), ATG7 (ab52472, Abcam), Numb (ab14140, Abcam)

1:1,000: LC3 (NB 100-2220, Novus), ATG16L1 (pAb PM040, MBL), Beclin (#3738S, Cell Signalling), VAMP3 (gift from A.A. Peden)

1:2,000: Actin (A2066,Sigma-Aldrich).

Notch1 on western blot shows Notch NTMD (125 kDa). It is the form which is cleaved during maturation at the plasma membrane. The NICD (activated Notch1) antibody detects VLLSRKRRRQHGQC, a sequence, which is not accessible in the uncleaved form. It is exposed after S1 cleavage. The protein is detected at 80 kDa.

### Biotinylation assay

HEK cells were incubated in 0.5–1 mg ml^−1^ NHS-LC-Biotin in Biotinylation buffer (1 mM MgCl_2_, 2 mM CaCl_2_, 150 mM NaCl) for 60–90 min with horizontal motion at 4 °C. After labelling, the plates were washed with the quenching buffer (1 mM MgCl_2_, 0.1 mM CaCl_2_, 100 mM glycine) twice for 10 min at 4 °C. Then the cells were lysed in RIPA buffer (150 mM NaCl, 50 mM Tris-HCl (pH 7.4), 5 mM EDTA, 1% Triton X-100, 0.5% deoxycholate, and 0.1% SDS, cocktail of protease and phosphatase inhibitors) for 30 min on ice. After centrifuging the lysates at 16,000*g* for 10 min at 4 °C, the protein concentration was determined using the Bradford method following the protocol of the Bio-Rad assay. 10 μl of each sample was kept for input control. 50 μl of Streptavidin-agarose beads (previously washed with PBS/RIPA) were added to each sample and incubated for 2 h at 4 °C (orbital/horizontal motion). Then the beads were washed twice with RIPA buffer and once with PBS. Finally, 20–30 μl of 2 × laemmli buffer was added to the beads and the samples were incubated at 95 °C for 5 min.

### Immunofluorescence

For immunocytochemistry, the cells were incubated in 200 nM Rapamycin (rap), 400 nM Bafilomycin A1 (baf), 100 mM Trehalose (treh) for 8 h or starved with HBSS for 2 h (1 × wash with HBSS before incubation for 2 h).

The cells were fixed for 3 min with ice cold methanol or for 10 min with 4% paraformaldehyde (PFA). Concentration of the primary and secondary antibodies are described below. The mounting solution was from Molecular Probes.

*Dilution of primary antibodies*. 1:50: Notch1 ms (ab44986, Abcam)

1:100: Notch1 rb (ab52627, Abcam), NICD (ab8925, Abcam), Hes1 (ab71559, Abcam), Pax6 (ab5790, Abcam), EEA1 (ab70521, Abcam), Dll1(ab76655, Abcam), ATG16L1 (Cell Signalling)

1:200: ATG9 (ab108338, Abcam)

1:300: LC3 (clone 5F10, Nanotools)

1:500: Tbr1 (ab31940, Abcam), Tbr2 (ab23345, Abcam), Nestin (ab6142. Abcam), 3β-tubulin (ab7751, Abcam)

1:800: AP2 (ab52222, Abcam)

The secondary antibodies Alexa 488, 568, 594 or 647 goat anti-mouse or goat anti-rabbit were obtained from Molecular Probes and used at 1:500.

Imaging was conducted with LSM710 Zeiss confocal with 63 × oil-immersion lense. The colocalization was measured using Volocity software for Mander's coefficient.

### Counting and quantification of vesicles

Single, double and triple positive vesicles (stained for Notch1/LC3/ATG16L1/ATG9/EEA1) were quantified by counting. The ratio of x-positive cells to total y-positive cells was formed.

### Proximity ligation assay

HEK cells were starved for 2 h in HBSS or left in basal state and proximity ligation assay was carried out according to the manufactorer's protocol (Sigma-Aldrich). Primary antibodies were used at the same concentration and time as in the immunofluorescence.

### Surface internalization assay

HEK cells were incubated on ice for 5 min and then incubated with ms Notch1 antibody on ice for 15 min. After three PBS washes, the cells were returned to a 37 °C incubator for 30 min. Then the cells were fixed by adding 1:1 warm 2% PFA to the PBS. Cells were incubated for 10 min at 37 °C for fixation and immunostaining, as described above. 1% BSA was used as the blocking solution and the washes were carried out with 1 × PBS. As a negative control, no Notch1 antibody, but Alexa 488, was added.

### Primary neuronal culture

All mouse experiments were performed under appropriate Home Office Licences (Procedure Project Licence number: 80/2593). For cortical cultures, embryos were harvested at E16.5. The neurons of two brains can be distributed on one six-well plate. Wells were coated with poly-ornithine (20 μg ml^−1^). The embryos were taken out of the uterus with scissors and the heads were removed. Heads were washed 3–4 times in PBS/0.6% glucose, the brain was removed and cut into two hemispheres, the meninges were carefully removed in each semicortex and the the striatum was taken out. Then the cortex was chopped into small pieces and transferred to 5 ml of PBS/0.6% glucose, and homogenized by pipetting up and down through a fine glass pipette. The suspension was left for 5 min to settle large clumps and the supernatant was centrifuged for 5 min at 149*g* 4 °C. 24 ml media was added to the cell pellet and 1 ml per well was seeded. After 30 min, the media was replaced. Media was then changed every 3 days. The cortical cells were used for experiments after 1 week of culturing.

### Brain lysates

5-week-old wild-type BL6/BC122 or Atg16L1 hypomorph mice (from Dr Herbert Virgin) were sacrificed. Half of the brain was homogenized in 1 ml of extraction buffer (0.5% Triton X-100, 50 mM Tris-HCl, 1 × Protease inhibitor) on ice with a glass hand-held homogenizer. The homogenate was centrifuged for 10 min at 16,000*g* and 4 °C. The supernatant was recentrifuged under the same conditions.

### Umbilical cord perfusion of embryos

The embryos were taken out carefully, without disconnecting them from the mother. 1 × PBS was pumped into the placenta of the embryo, followed by 4% PFA injection. After the blood was cleaned out, the embryos were incubated in 4% PFA overnight.

### Immunohistochemistry

Brains of 5 weeks old mice were obtained after perfusion and sectioned in 10 μm thin slices on +P slides. Guts of same age mice were isolated and incubated in 4% PFA overnight, following 30% sucrose incubation before sectioning into 10 μm thin slices. After drying overnight at 37 °C and the tissue was refixed with 4% PFA, permeabilized and blocked with 0.1% Triton-X in 3% goat serum for 1 h. The following steps follow the same protocol as for immunofluorescence, except that the primary antibody was applied over night at 4 °C without shaking.

*Dilution primary antibodies*. 1:25: Notch1 (ab44986, Abcam)

1:50: BrdU (ab6326, Abcam)

1:100: NICD (ab8925, Abcam), Nestin (ab6142, Abcam)

1:250: Grem1 (SAB1301532, Sigma)

1:300: 3β-tubulin (ab31940, Abcam), Doublecortin (ab18723, Abcam)

The secondary antibodies Alexa 488, 568 goat anti-mouse or goat anti-rabbit were obtained from Molecular Probes and used at 1:500 for 2 h.

Imaging was conducted with LSM710 Zeiss confocal with 20 × air lense.

### Image J quantification

For the measuring the intensity of the developmental marker stainings in primary neurons, the fluorescence intensity was measured for three fields from three images of one sample and the average was taken. The s.e.m. was formed between the averages of the three independent experiments. To determine the size of the neuronal regions in the wild-type and Atg16L1 hypomorph brain slices, the area of the 3β-tub and Dbx-positive region was measured. The 3β-tub or Dbx positive area was divided by the DAPI-positive area to normalize against the slice size. For the Nestin- (stem cell marker) positive brain slices, the Nestin intensity over the whole slice was divided by the DAPI intensity (Since Nestin staining does not mark a defined region). Five slices was included into the measurement for each embryo. All data was analysed blinded.

### DAPT drug treatment

Increasing concentrations of DAPT (Sigma) from 1 to 10^5^ nM were added to cultures of E16.5 wild-type or Atg16L1 hypomorph primary neurons 4 days after isolation and incubated for 2 days.

For the treatment of mice, 20 mg kg^−1^ DAPT was subjected via intraperitoneal injection once daily for 6 successive days. On day 6, the mice were also injected with 50 mg kg^−1^ BrdU and for 5 successive days with BrdU only. The mice were perfused on day 12 and frozen sections cut. BrdU staining was carried out according to the manufacturer's protocol (Abcam).

### Bone marrow extraction

6 weeks old wild-type or Atg16L1 hypomorph mice were sacrificed for bone marrow extraction. After the isolation of the femur and tibia from the rest of the tissue, the ends of the bones were cut open and the bone marrow was extracted by syringes containing PBS/2% FBS solution. The cells were centrifuged at 21*g* at 4 °C, whereafter the supernatant was removed.

### Flow cytometry

After 30 min of blocking with PBS/2%FBS solution, primary antibodies were applied to the bone marrow cells for 45 min.

1.5:100: Sca1-phosphatidylethanolamine (122507, BioLegend), c-kit-APC-Cy7 (105825, BioLegend), Notch1 (ab52627, Abcam)

1:100: mouse HSC isolation cocktail (Stem cell technology)

Secondary antibody incubation was 15 min.

0.5:100: Streptavidin V500 (BD)

1:300: Alexa 488

Then 7AAD (life technologies) was added to the cells with a dilution of (1:1,000). The samples were analysed with the Fortessa 4 Flow cytometer and the graphs and statistics created with the program FlowJo.

The threshold for Notch1 was defined with the secondary staining-only population, set for <98% of the distribution as the negative population. This gating creates discrete valuables for the percentage of Notch1-positive cell in each sample from six sets of experiments, each containing a pair of wild-type and Atg16L1 hypomorph sample. The Atg16L1 hyp value was normalized against its wild-type pair and the statistics was performed on the six sets of experiments. For the discrete values a one-sample *t*-test was performed.

### Statistics

Significance levels for comparisons between two groups were determined with two-tailed *t*-test. *P≤0.05; **P≤0.01; ***P≤0.001. Error bars represent s.e.m.

### Full blot images

Full blot images are shown for important blots in Supplementary Fig. 12.

## Additional information

**How to cite this article:** Wu, X. *et al.* Autophagy regulates Notch degradation and modulates stem cell development and neurogenesis. *Nat. Commun.* 7:10533 doi: 10.1038/ncomms10533 (2016).

## Supplementary Material

Supplementary Information Supplementary Figures 1-12
