# Supplementary material for: Autophagy regulates Notch degradation and modulates stem cell development and neurogenesis
Source: Nat Commun. 2016 Feb 3;7:10533. doi: 10.1038/ncomms10533 (PMC4742842; doi:10.1038/ncomms10533)

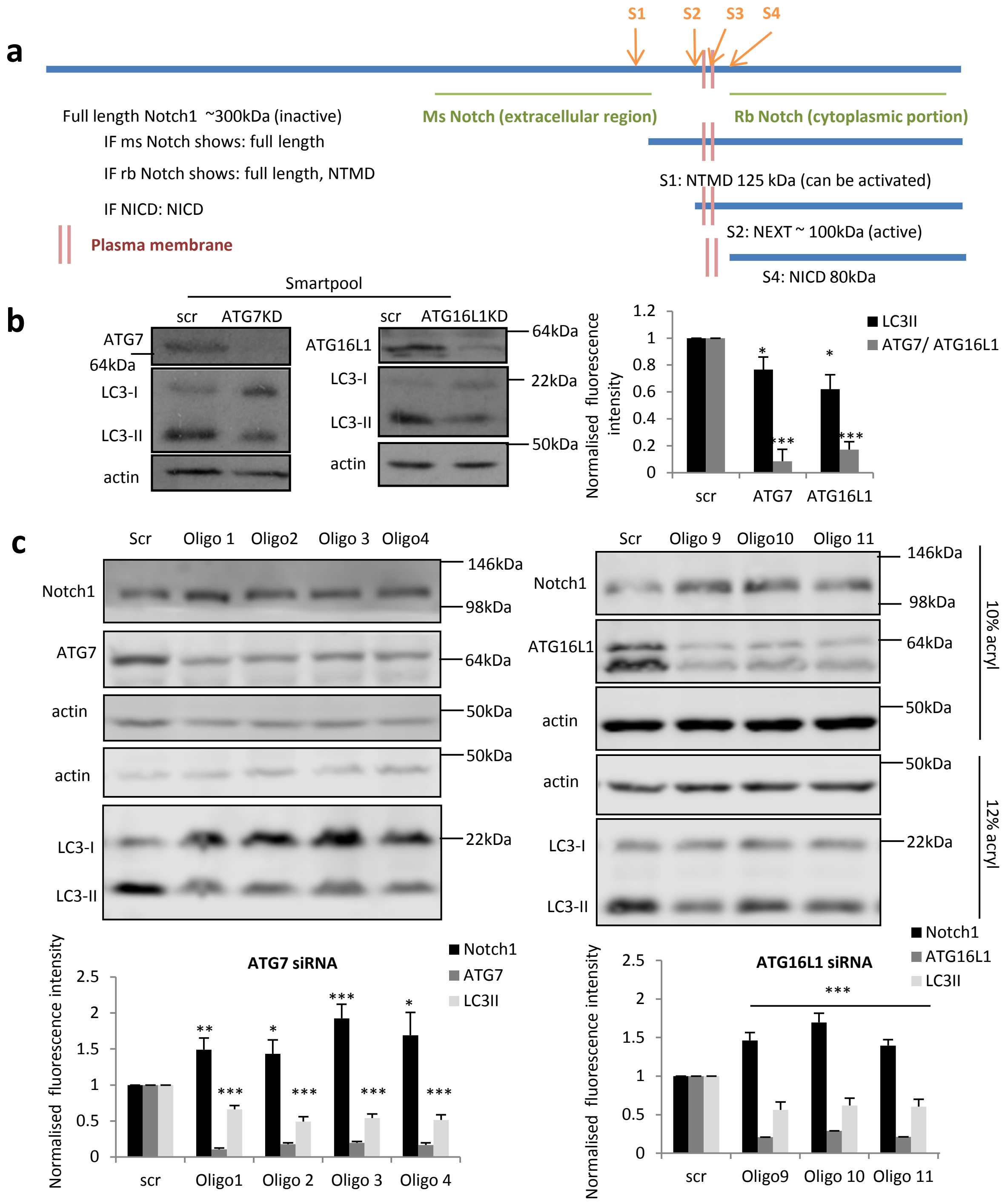

Supplementary Fig. 1.

(a) Notch1 is cleaved first (S1 cleavage) during maturation to generate a non-covalently bound NECD (Notch extracellular domain) and NTMD (Notch transmembrane domain), which can be activated later by the ligand. After ligand activation, Notch1 is cleaved (S2) into NEXT and then cleaved twice by the γ-secretase into the active form NICD (Notch intracellular domain), which translocates into the nucleus to activate transcription of Hes1. Green lines show the region used as antigen for creation of the mouse (Ms) and Rabbit (Rb) antibodies used in this manuscript, therefore mouse Notch1 antibody recognise full-length Notch1 only and rabbit Notch1 antibody recognises full-length and NTMD. The NICD antibody recognises an intracellular region, which was masked before S4 cleavage.

(b) Representative western blot showing the effect of ATG7, ATG16L1 Smartpool knock-down on LC3-II levels. HEK cells were transfected with scrambled (scr)/ ATG7/ ATG16L1 siRNA. Protein levels of ATG7, ATG16L1, and LC3-II were assessed by western blot along with actin as a loading control and protein levels were quantified relative to actin and normalised for the relevant control. * or ** or ***denotes $p < 0.05$ or $p < 0.01$ or $p < 0.001$ by paired t-test. n =3. Error bars = S.E.M.

(c) Representative western blot showing the effect of ATG7 or ATG16L1 knock-down with separate deconvoluted oligonucleotides on Notch1, ATG7/ ATG16L1 and LC3-II levels. HEK cells were transfected with scrambled (scr)/ ATG16L1 oligo 9-11/ ATG7 oligo 1-4 siRNA. Protein levels of Notch1, ATG16L1, ATG7, and LC3-II were assessed by western blot along with actin as a loading control and protein levels were quantified relative to actin and normalised for the relevant control. The level of Notch1/ ATG16L1/ ATG7/ LC3-II in the oligo knock-down conditions were compared with the respective protein levels in the scrambled condition. In the ATG7 knock-down experiment, the ATG7 and LC3-II levels were highly significantly different (***), when compared with the scrambled condition. In the ATG16L1 knock-down condition all the samples were highly significantly different (***) when compared to the scrambled condition. *or ** or ***denotes $p < 0.05$ $p < 0.01$ or $p < 0.001$ by paired t-test for protein levels in siRNA KD cells relative to scr conditions. $n = 3$. Error bars = S.E.M.

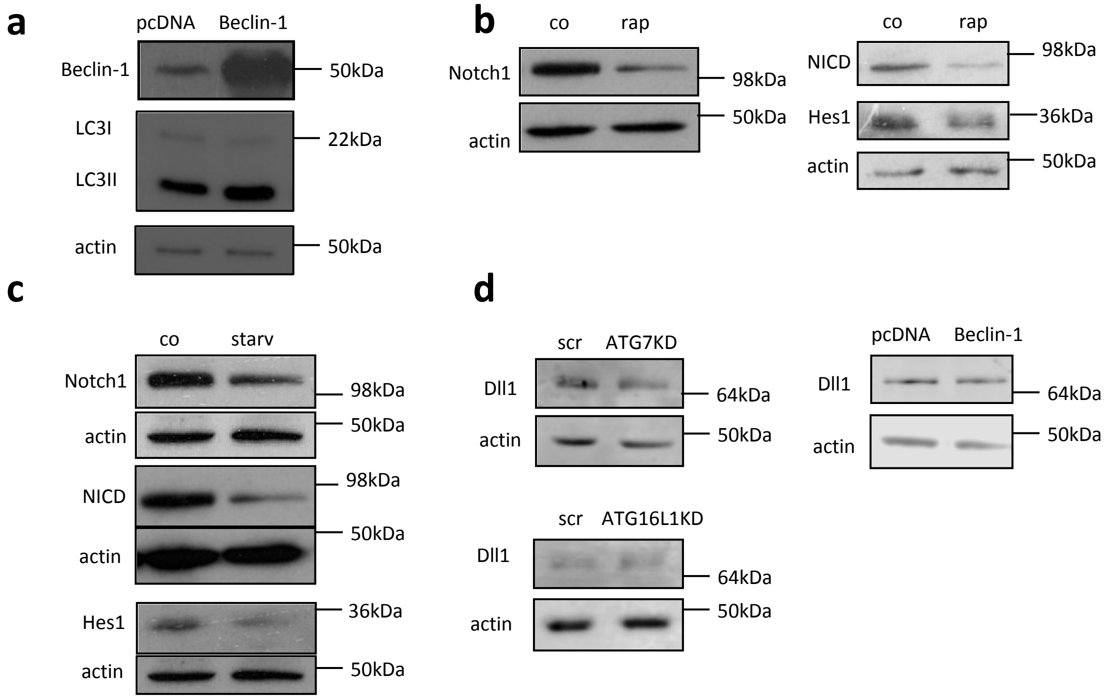

Supplementary Fig. 2.

(a) Representative western blot showing the effect of Beclin-1 overexpression on LC3-II levels. HEK cells were transfected with pcDNA/ Beclin-Flag constructs. Protein levels of Beclin-1 and LC3-II were assessed by western blot along with actin as a loading control.

(b) Representative western blot showing the effect of pharmacological autophagy modulation on Notch1, NICD and Hes1. HEK cells were treated with rapamycin (6 h). Protein levels of Notch1, NICD and Hes1 was assessed by western blot along with actin as a loading control.

(c) Representative western blot showing the effect of starvation on Notch1, NICD and Hes1. HEK cells were starved (2 h). Protein levels of Notch1, NICD and Hes1 was assessed by western blot along with actin as a loading control.

(d) Representative western blot showing the effect of ATG7, ATG16L1 knock-down and Beclin-1 overexpression on Dll1 level. HEK cells were transfected with scrambled (scr)/ ATG7/ ATG16L1 siRNA or pcDNA/ Beclin-Flag construct. Protein levels of Dll1 were assessed by western blot, using actin as a loading control.

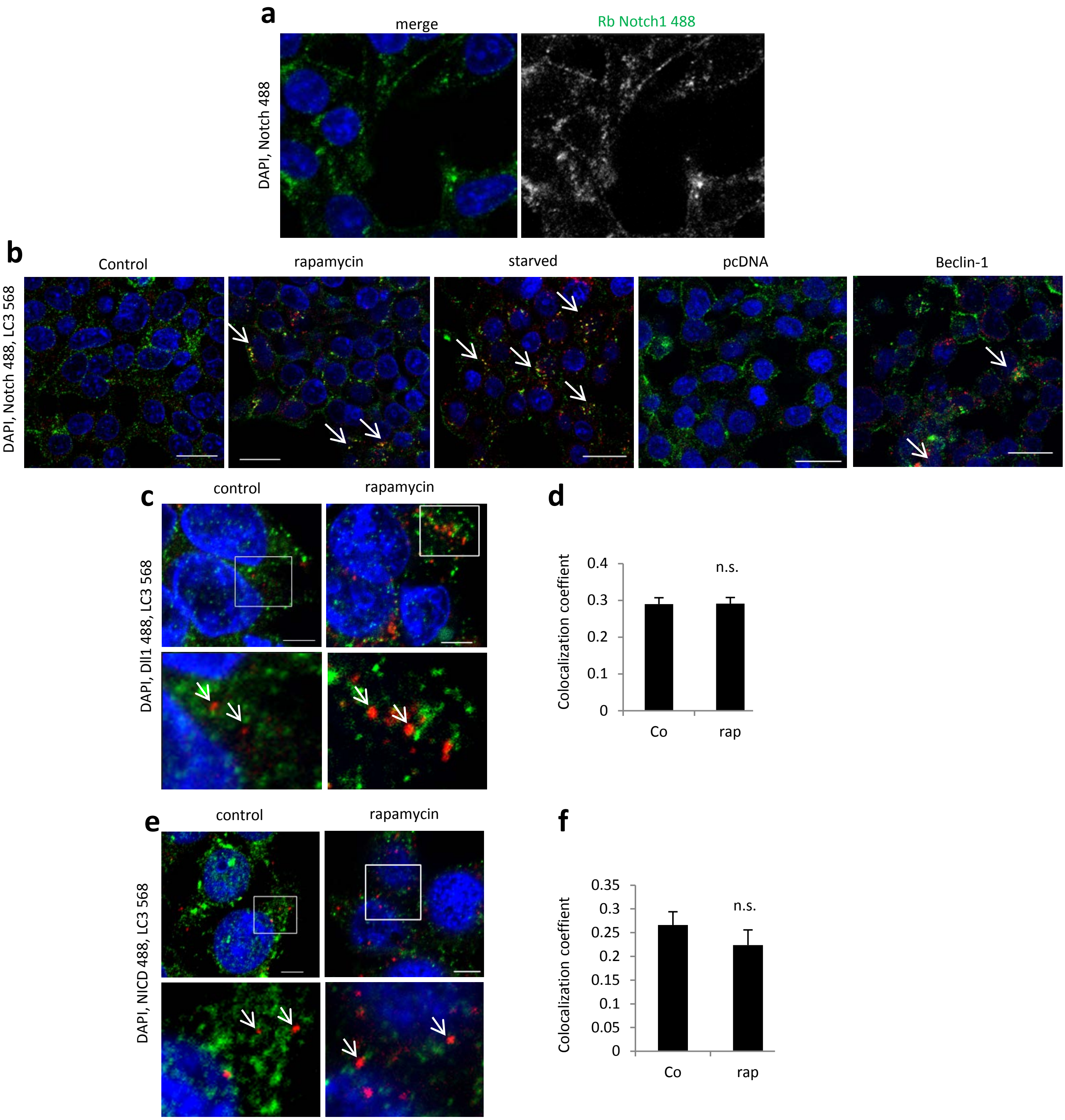

Supplementary Fig. 3.

(a) Notch1 staining at the plasma membrane. The focus of the confocal plane was fixed at the plasma membrane to visualise Notch1 staining (green). The right panel shows Notch1 staining only.

(b) HEK cells starved with HBSS (2 h) or treated with DMSO (control) /rapamycin or transfected with pcDNA or Beclin-Flag (2 days). Cells were immunostained for Notch1 (green) and LC3 (red) and stained with DAPI (blue). The arrows show LC3 and Notch1 colocalization. Scale bar = 20µm

(c) Effect of pharmacological autophagy modulation on the colocalization of Dll1 and LC3. HEK cells were treated with DMSO (control) /rapamycin for 8 h and immunostained for Dll1 (green) and LC3 (red). The arrows show LC3 vesicles. Scale bar = 5 µm. Lower panels show an enlargement of the area in the white box.

(d) Quantification of confocal images for Mander's colocalization between Dll1 and LC3 in control cells (Co) or cells treated with rapamycin (rap). n.s. denotes not significant by unpaired t-test. n = 3. Error bars = S.E.M.
(e) Effect of pharmacological autophagy modulation on the colocalization of NICD and LC3. HEK cells were treated with DMSO (control) /rapamycin for 8 h and stained for NICD (green) and LC3 (red). The arrows show LC3 vesicles. Scale bar = 5 μm
(f) Quantification of confocal images for Mander's colocalization between NICD and LC3 in control cells (Co) or cells treated with rapamycin (rap). n.s. denotes not significant by unpaired t-test. n = 3. Error bars = S.E.M.

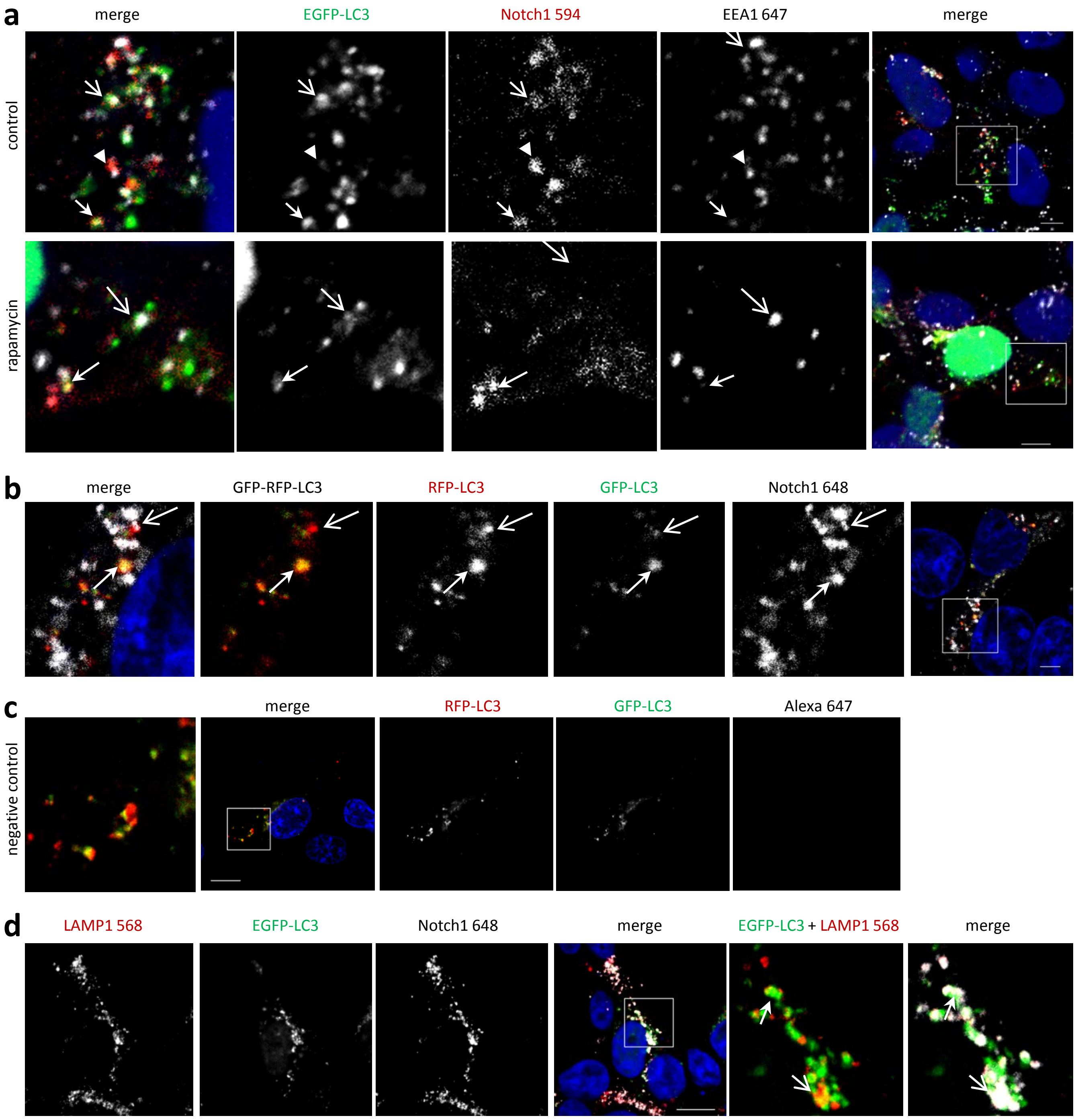

Supplementary Fig. 4.

(a) Additional representative images supporting Fig. 3b: Effect of autophagy induction on Notch1 colocalisation with markers for the autophagy (pEGFP-LC3) or endocytic (EEA1) pathway. HEK cells were transfected with pEGFP-LC3 and then treated with DMSO (control) or rapamycin and immunostained for Notch1 (red) and EEA1 (white) and stained for DAPI (blue). shows Notch1-EEA1 only colocalization, indicates LC3-Notch1 only colocalization, → indicates triple colocalization. Scale bar = 5 µm.

(b) Additional representative images supporting Fig. 3g: Confocal images showing colocalization between Notch1 and markers of autophagosomes. HEK cells were transfected with RFP-GFP-LC3 and stained for Notch1. indicates Notch1 colocalization with RFP- and GFP-positive structures. → shows Notch1 colocalization with RFP-positive and GFP-negative structures. Scale bar = 5 µm.

(c) Negative control to Fig. 3g: HEK cells were transfected with RFP-GFP-LC3 and stained only with the secondary Alexa 647. Scale bar = 5 µm.

(d) Additional representative images supporting Fig. 3i: Confocal images showing colocalization between Notch1 and markers of autophagosomes and lysosomes. HEK cells were transfected with pEGFP-LC3 and stained against LAMP1 and Notch1. shows Notch1 colocalization with GFP-positive, LAMP1-negative vesicles. → indicates triple positive structures. Scale bar = 5 µm.

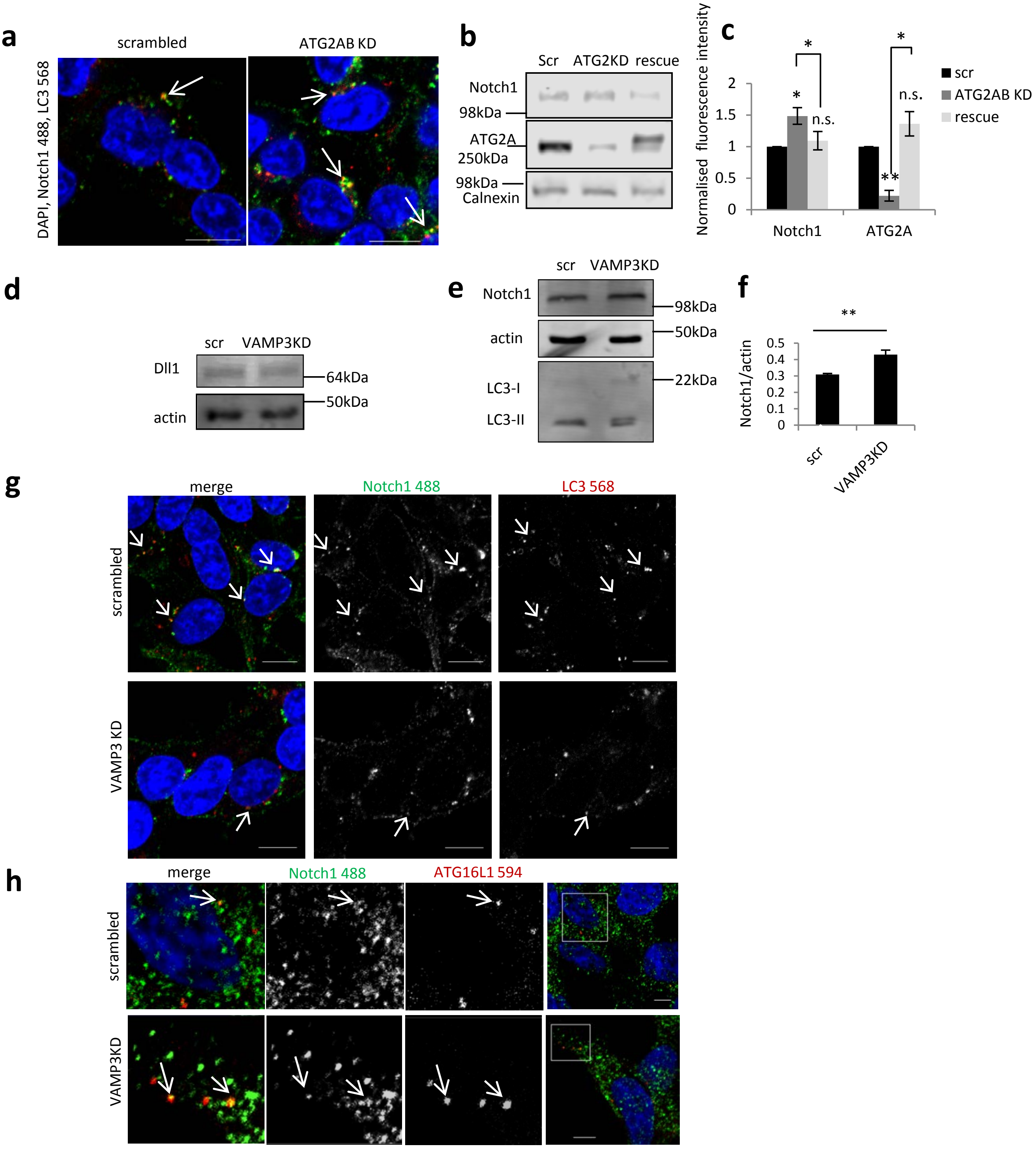

Supplementary Fig. 5.

(a) HEK cells showing the effect of inhibition of autophagy pathway components downstream of LC3 (ATG2A and ATG2B) on the colocalization of Notch1 (green) with LC3 (red). HEK cells were transfected with scrambled siRNA (scr) or ATG2A/B siRNA. The arrows show LC3 and Notch1 colocalization. Scale bar = 10 µm

(b) Representative western blot showing the effect of ATG2A/B knock-down and rescue on the level of Notch1. HEK cells were transfected with scrambled (scr)/ ATG2A/B oligos and ATG2A/B oligos + siRNA resistant ATG2A-GFP construct. Protein levels of Notch1 and ATG2A/ ATG2A-GFP were assessed by western blot along with calnexin as a loading control.

(c) Quantification of western blots for Notch1 and ATG2A/ ATG2A-GFP, relative to calnexin and normalised for the relevant control. * or **denotes $p < 0.05$ or $p < 0.01$ by paired t-test. n.s. denotes not significant. $n = 3$. Error bars = S.E.M.
(d) Representative western blot showing the effect of VAMP3 knock-down on the level of Dll11. HEK cells were transfected with scrambled (scr)/ VAMP3 oligo. Protein levels of Dll1 were assessed by western blot along with actin as a loading control.
(e) Representative western blot showing the effect in HEK cells of VAMP3 siRNA on Notch1 and LC3-II.
(f) Quantification of Notch1 protein level in scrambled siRNA (scr) and VAMP3 siRNA transfected samples. **denotes $p <0.01$ by unpaired t-test. $n = 3$.
(g) HEK cells showing the effect of inhibition of autophagy pathway components upstream of LC3 (VAMP3) on the colocalization of Notch1 with LC3. HEK cells were transfected with scrambled siRNA (scr) or VAMP3 siRNA. The arrows show LC3 and Notch1 colocalization. Scale bar = 10 µm
(h) Additional image supporting Fig. 4h: Effect of inhibition of autophagy pathway components downstream of ATG16L1 (VAMP3) on the colocalization of Notch1 with ATG16L1. HEK cells were transfected with scrambled siRNA (scr) or VAMP3 siRNA. The arrows show ATG16L1 and Notch1 colocalization. Scale bar = 5 µm

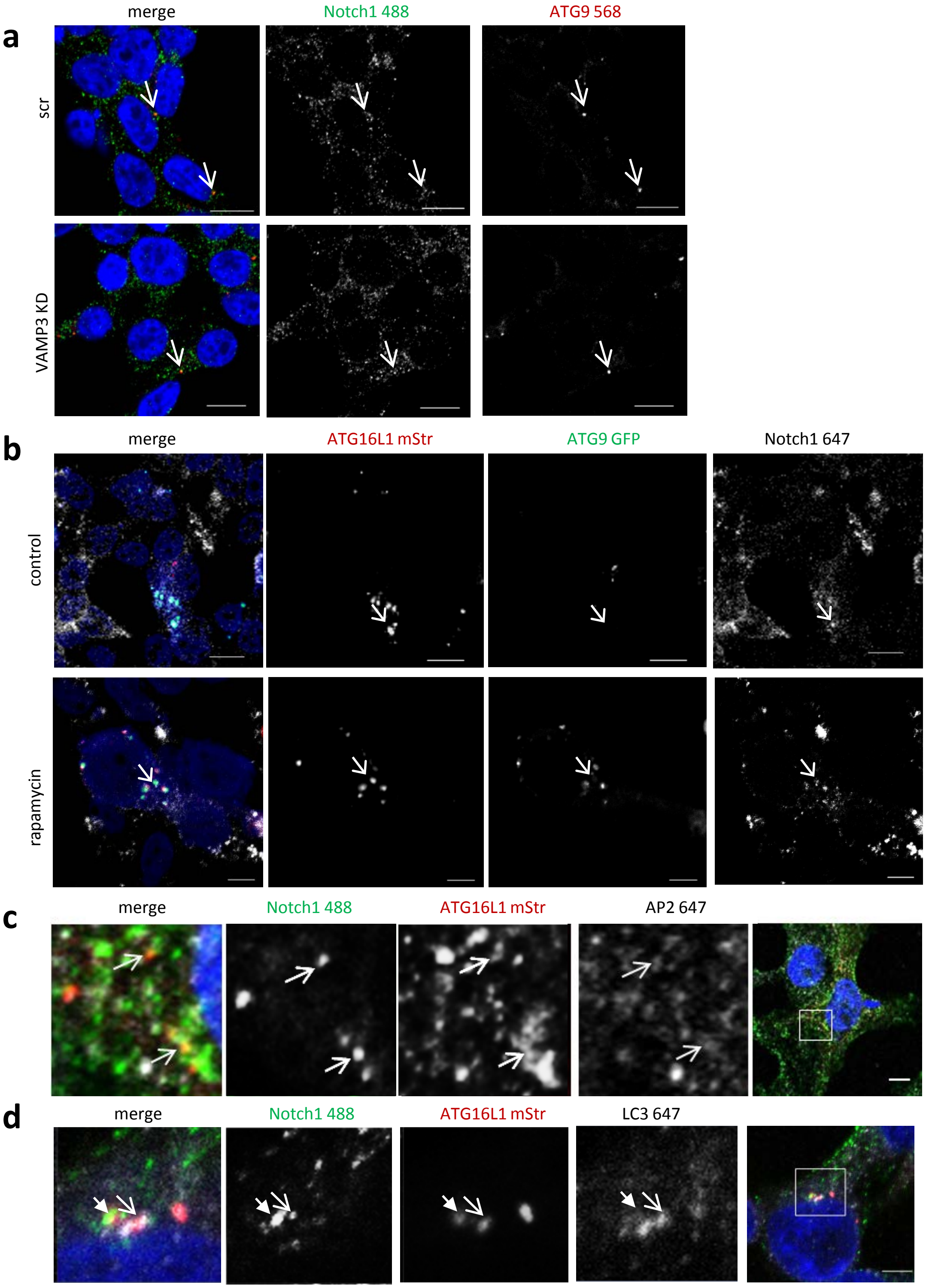

Supplementary Fig. 6.

(a) HEK cells showing the effect of inhibition of autophagy pathway component downstream of ATG9 (VAMP3) on the colocalization of Notch1 with ATG9. HEK cells were transfected with scrambled siRNA (scr) or VAMP3 siRNA. The arrows show ATG9 and Notch1 colocalization. Scale bar = 10 µm.

(b) HEK cells showing the effect of autophagy on Notch1 colocalization with autophagy precursors marking two distinct autophagy initiation pathways (ATG16- or ATG9- positive vesicles). HEK cells were transfected with ATG9-pEGFP (ATG9-GFP) and pmStrawberry-ATG16L1 (ATG16 mStr), treated with DMSO/ rap for 8 h. → shows Atg16-Notch1 only colocalization. scale bar = 10 µm

(c) Colocalization of Notch1 with markers of ATG16L1 endocytosis. HEK cells were transfected with pStrawberry-ATG16L1 and stained for Notch1 and AP2. The arrows show ATG16L1, Notch1 and AP2 colocalization. Scale bar = 5 µm
(d) Effect of autophagy induction on Notch1 and ATG16L1 colocalization. HEK cells were transfected with pStrawberry-ATG16L1 and stained for Notch1 and LC3. Small, filled arrowhead indicates Notch1 and ATG16 colocalization in the absence of LC3, → indicates Notch1, ATG16L1 and LC3 colocalization. Scale bar = 5 µm.

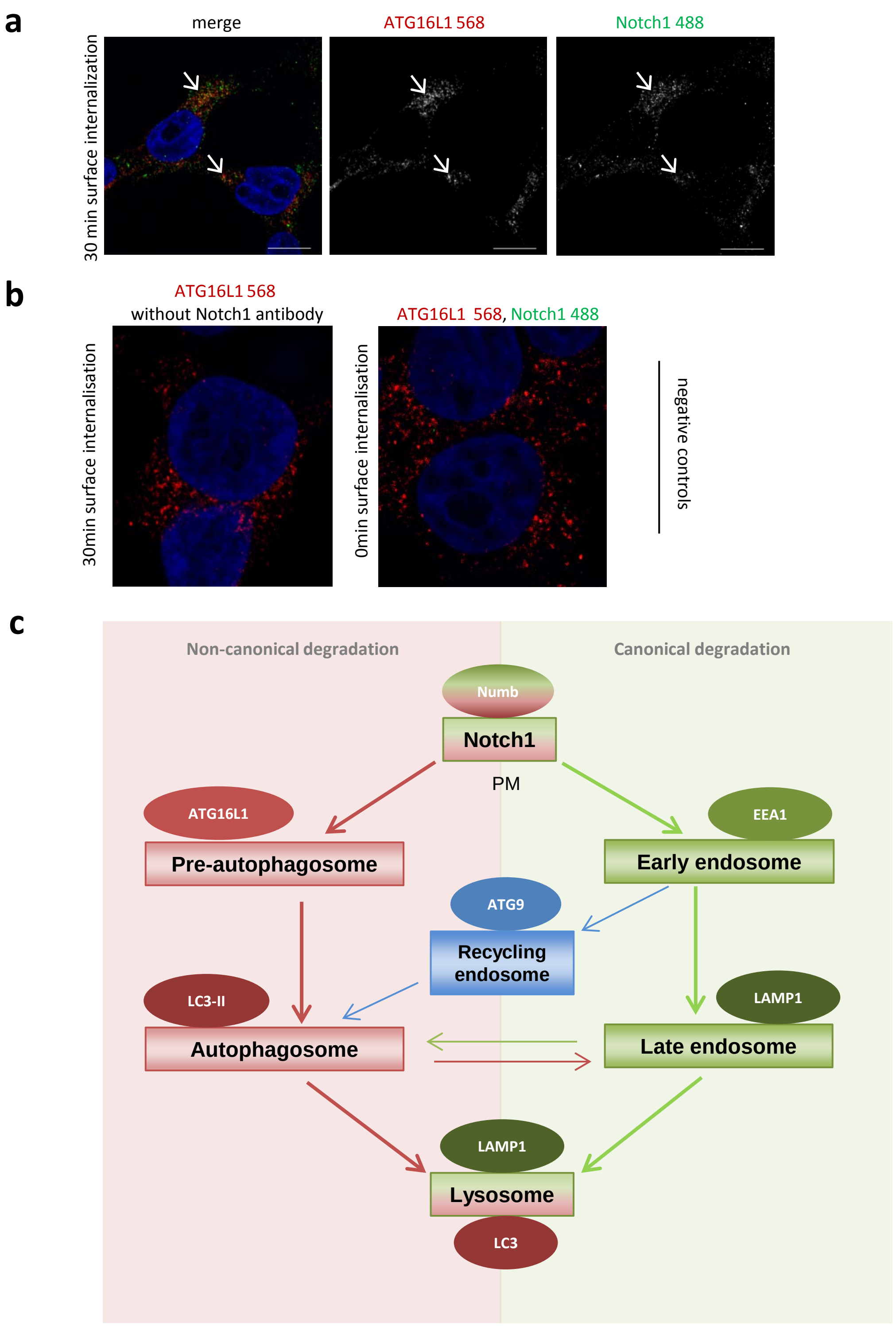

Supplementary Fig. 7.

(a) Additional image supporting Fig. 5d: Colocalization of Notch1 and ATG16L1 after surface internalisation for 30 min. HEK cells were incubated with Notch1 antibody on ice and then for 30 min at 37ºC to allow internalisation of labelled Notch1 cell surface receptors. Arrows indicate Notch1 (Alexa 488) and ATG16L1 (Alexa 568) double-positive structures. The negative control lacks incubation with Notch1 antibody. Scale bar = 10 µm

(b) Negative controls for the surface internalization experiment of Notch1 shown in Fig. 5d: The negative control in the right panel shows 0 min internalization and the left panel is without Notch1 primary antibody.

(c) Schematic diagram of Notch1 degradation by autophagy and endocytosis. Notch1 is internalised from the plasma membrane (PM) – canonical pathway via endocytosis indicated by green arrows. Notch1 is known to be internalised by the early endosome (EEA1). The degradation route goes to the late endosome, which then fuses with the lysosome. Interaction between the endocytosis and autophagy pathways could be predicted as demonstrated by black arrows. EEA1 might meet the recycling endosome and fuse with ATG9 vesicles and then autophagosomes. However, we propose a new route for Notch1 internalisation and degradation via the autophagy pathway indicated by red arrows. Notch1 is taken up from the plasma membrane by ATG16L1 vesicles through clathrin mediated uptake (adaptor AP2). It goes directly into autophagosomes and then autophagosomes fuse with lysosomes for degradation of their contents.

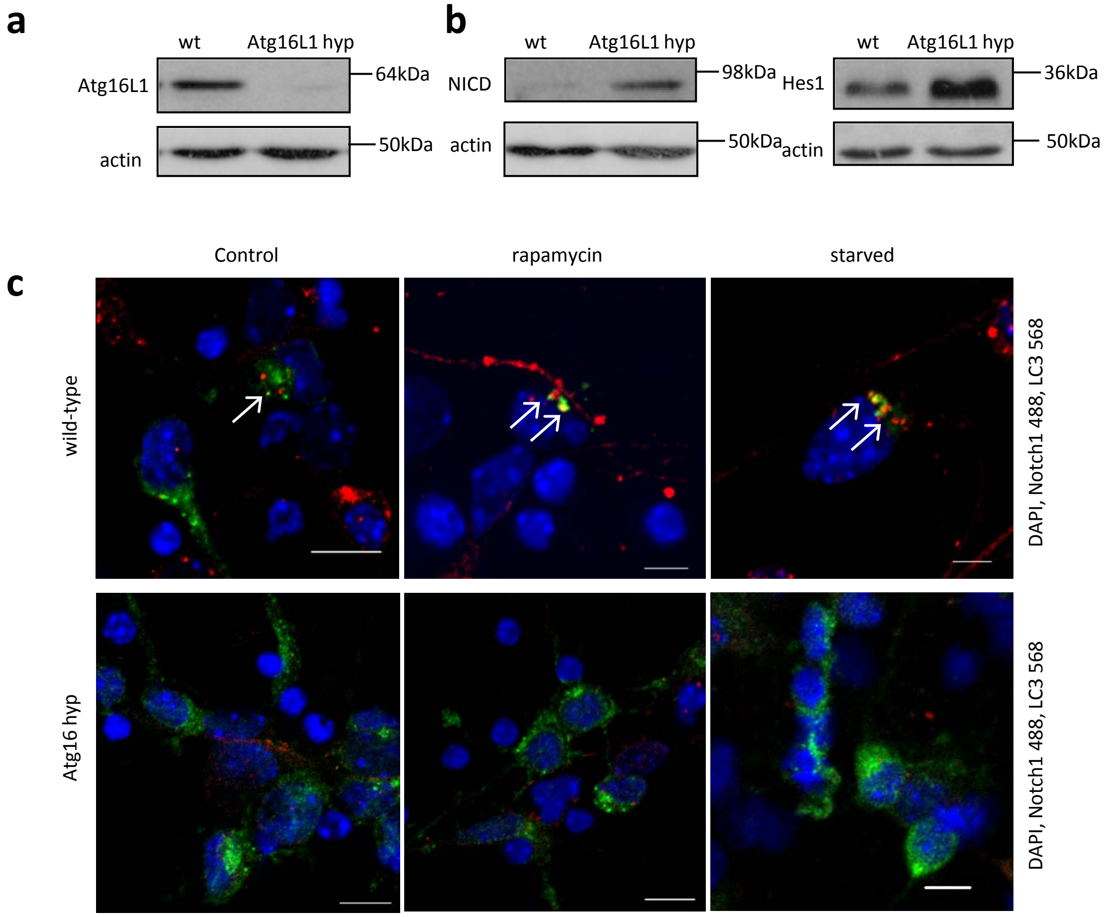

Supplementary Fig. 8.

(a) Western blots showing levels of Atg16L1 in wild-type and Atg16L1 hypomorph (Atg16hyp) brain lysates of 5 week old mice. Images are representative of data obtained from lysates from 3 wild-type (wt) and 3 Atg16L1 hypomorph (Atg16hyp) animals.

(b) Western blots showing levels of NICD and Hes1 in wild-type and Atg16L1 hypomorph (Atg16hyp) brain lysates of 5 week old mice. Images are representative of data obtained from lysates from 3 wild-type (wt) and 3 Atg16L1 hypomorph (Atg16hyp) animals.

(c) Additional image supporting Fig. 6e: Effect of pharmacological autophagy modulation on the colocalization of Notch1 with LC3 in wild-type (E15.5) primary cortical neurons. Neurons were treated with DMSO (Control), starved with HBSS for 2 h or treated with rapamycin (8 h). The arrows denote Notch1 (green) and LC3 (red) colocalization. Scale bar = 10 µm

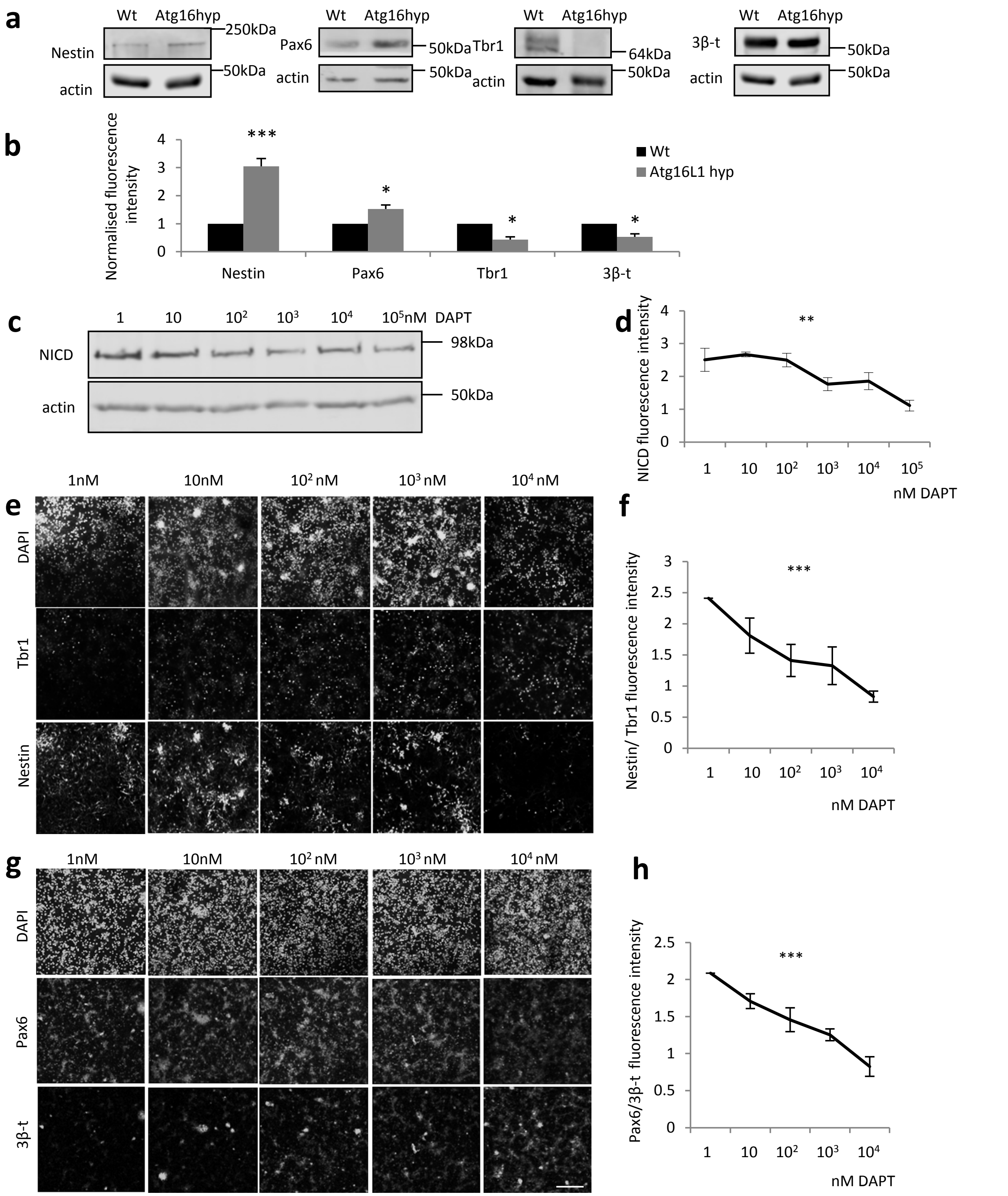

Supplementary Fig. 9.

(a) Representative western blots showing the levels of Nestin, Pax6, Tbr1 and 3β-tubulin along with actin as a loading control in cortical primary neuron lysates from E16.5 wild-type mice compared to Atg16L1 hypomorph mice.

(b) Quantification of western blots for Nestin, Pax6, Tbr1 and 3β-tubulin, relative to actin and normalised for the relevant control. * or ***denotes $p < 0.05$ or $p < 0.001$ by paired t-test. n = 3. Error bars = S.E.M.

(c) Representative western blot showing dose dependent effect of DAPT treatment on NICD level. Cortical primary neuron lysates from E15.5 Atg16L1 hyp mice were treated with increasing concentration of DAPT.

(d) Quantification of NICD protein level from western blots as shown in c. ** denotes $p < 0.01$ by Anova. n = 3. Error bars = S.E.M.
(e) Effect of DAPT treatment on the differentiation of wild-type neurons. Cortical primary neurons from E15.5 wild-type mice were treated with increasing concentration of DAPT and stained for Tbr1 and Nestin and nuclei were stained with DAPI (blue). Scale bar= 200 µm.
(f) Quantification of fluorescence intensity from immunostained primary neurons as shown in e. *** denotes $p < 0.001$ by Anova. n = 3. Error bars = S.E.M.
(g) Effect of DAPT treatment on the differentiation of wild-type neurons. Cortical primary neurons from E15.5 wild-type mice were treated with increasing concentration of DAPT and stained for Pax6 (green) and 3βtub (red) and nuclei were stained with DAPI (blue). Scale bar= 200 µm.
(h) Quantification of fluorescence intensity from immunostained primary neurons as shown in g. *** denotes $p < 0.001$ by Anova. n = 3. Error bars = S.E.M.

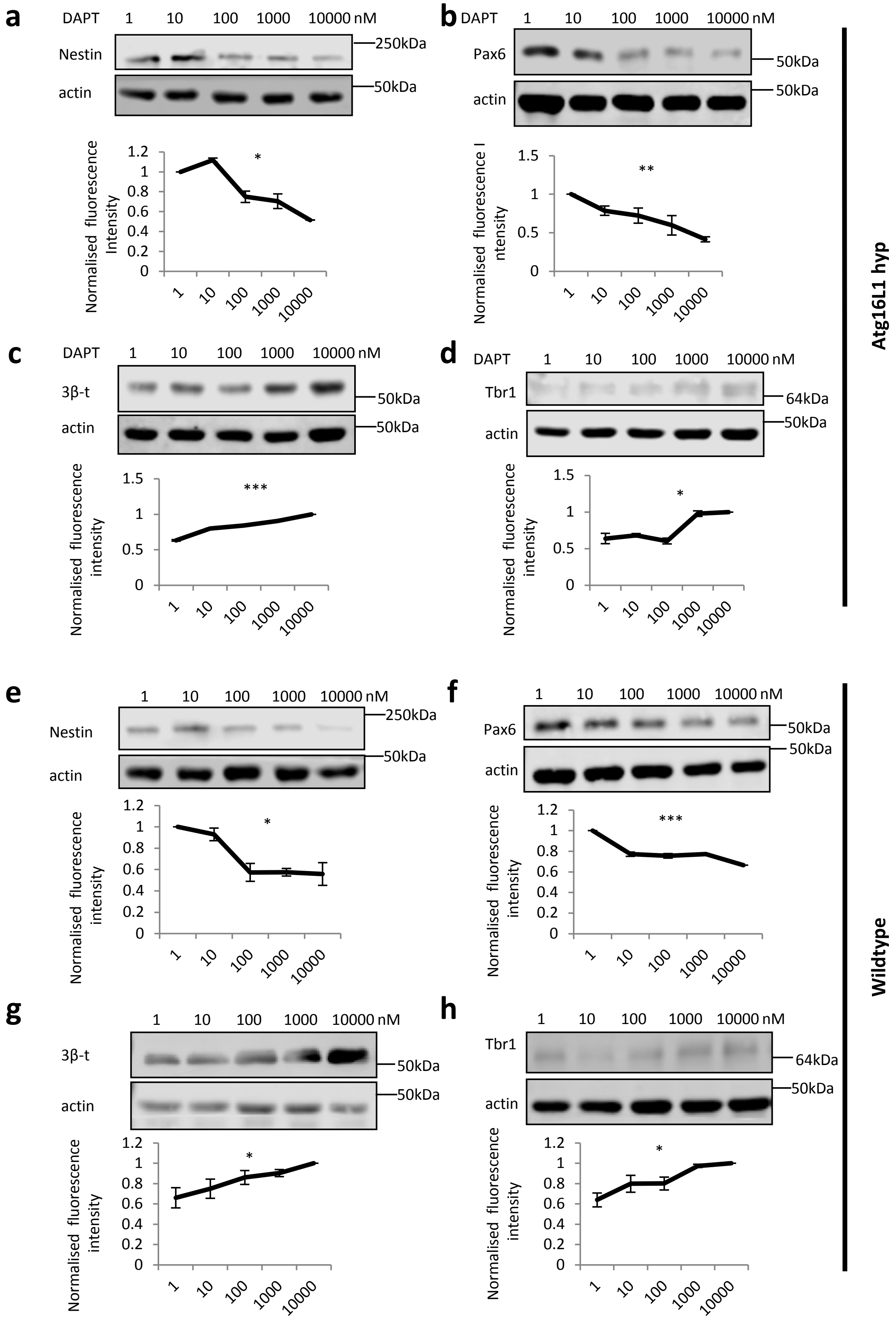

a
DAPT 1 10 100 1000 10000 nM
Nestin
actin
250kDa
50kDa
Normalised fluorescence Intensity
b
DAPT 1 10 100 1000 10000 nM
Pax6
actin
50kDa
Normalised fluorescence Intensity
c
DAPT 1 10 100 1000 10000 nM
3β-t
actin
50kDa
Normalised fluorescence intensity
d
DAPT 1 10 100 1000 10000 nM
Tbr1
actin
64kDa
50kDa
Normalised fluorescence intensity
Atg16L1 hyp
e
1 10 100 1000 10000 nM
Nestin
actin
250kDa
50kDa
Normalised fluorescence intensity
f
1 10 100 1000 10000 nM
Pax6
actin
50kDa
Normalised fluorescence intensity
g
1 10 100 1000 10000 nM
3β-t
actin
50kDa
Normalised fluorescence intensity
h
1 10 100 1000 10000 nM
Tbr1
actin
64kDa
50kDa
Normalised fluorescence intensity
Wildtype

Supplementary Fig. 10.

(a) - (d) Effect of DAPT treatment on the differentiation of Atg16L1 hypomorph neurons. Cortical primary neurons from E15.5 Atg16L1 hypomorph mice were treated with increasing concentration of DAPT and (a) Nestin/ (b) Pax6/ (c) Tbr1/ (d) 3β-tubulin and actin levels are shown by western blotting, as well as quantified relative to actin and normalised for the relevant control. * or ** or *** denotes $p < 0.05$ or $p < 0.01$ or $p < 0.001$ by Anova. n = 3. Error bars = S.E.M.

(e) - (h) Effect of DAPT treatment on the differentiation of wild-type neurons. Cortical primary neurons from E15.5 wild-type mice were treated with increasing concentration of DAPT and (e) Nestin/ (f) Pax6/ (g) Tbr1/ (h) 3β-tubulin and actin levels are shown by western blotting, as well as quantified relative to actin and normalised for the relevant control. * or *** denotes $p < 0.05$ or $p < 0.001$ by Anova. n = 3. Error bars = S.E.M.

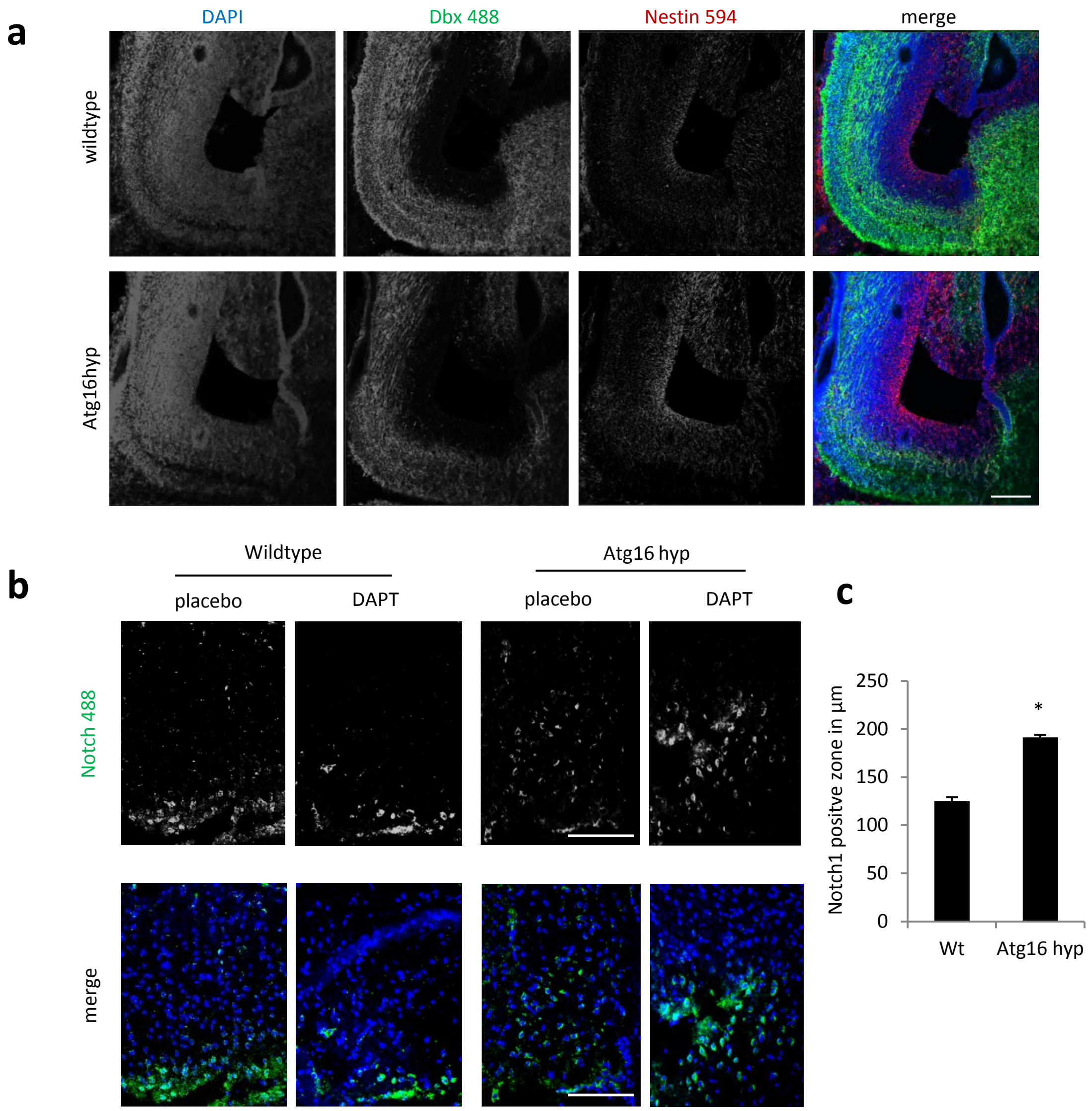

Supplementary Fig. 11.
(a) Nestin and Dbx immunostaining in E15.5 wild-type and Atg16L1 hypomorph (Atg16hyp) brains. Scale bar = 50 µm for all images in the panel.
(b) Notch1 and DAPI immunostaining in 9-11 months wild-type and Atg16L1 hypomorph mice brains. Scale bar = 50 µm is valid for all panels.
(c) Quantification of Notch1-positive zone wild-type (wt) and Atg16L1 hypomorph (Atg16hyp) brain slices. *denotes p <0.05 by unpaired t-test. n=3. Error bars = S.E.M.

Supplementary Figure 12

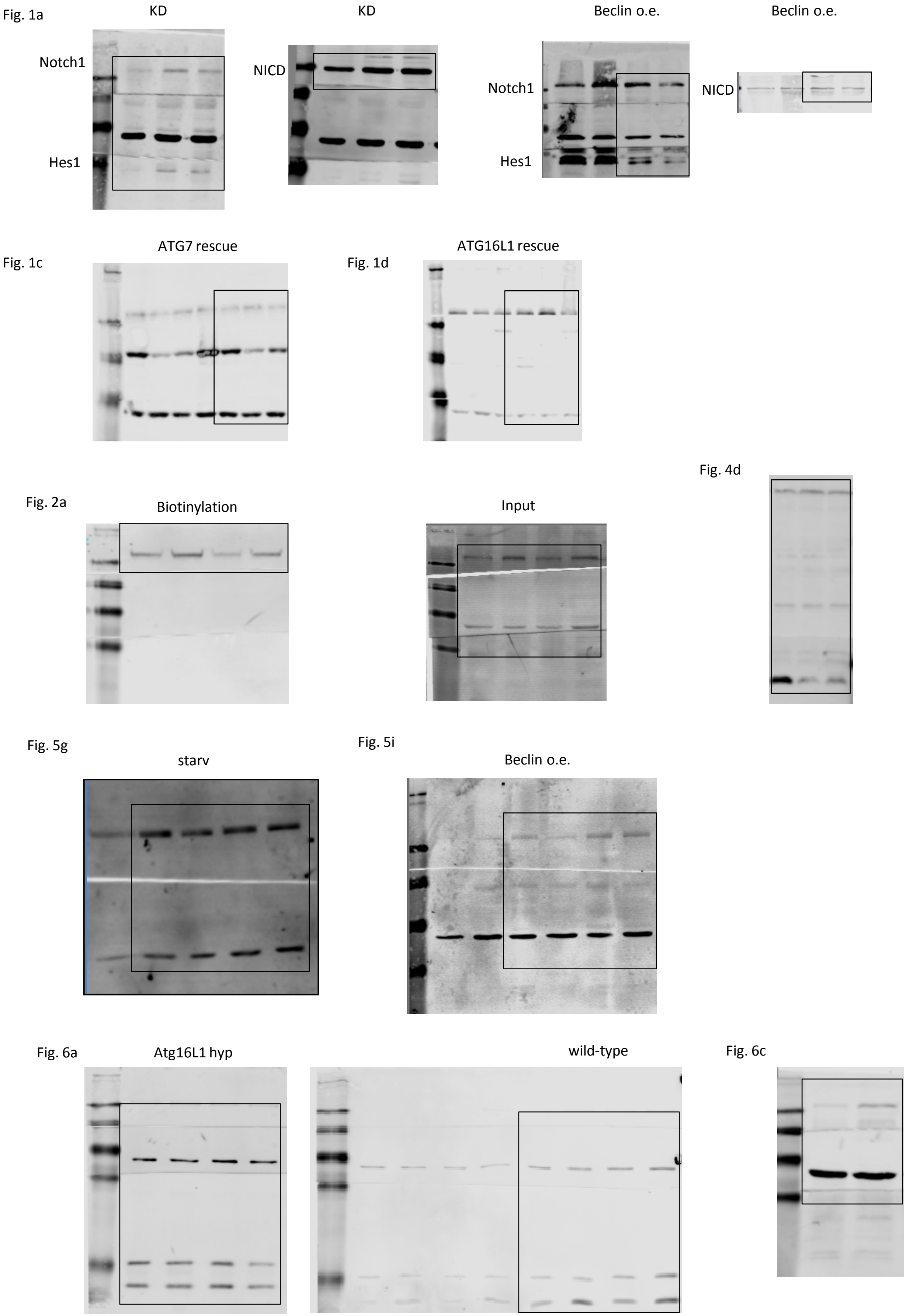

Supplement: Supplementary Information — Supplementary Figures 1-12 [file ncomms10533-s1.pdf]
